# Haptotactic Motion of Multivalent Vesicles Along Ligand-Density Gradients

**DOI:** 10.1021/acs.langmuir.5c00494

**Published:** 2025-04-29

**Authors:** Hannah Sleath, Bortolo M. Mognetti, Yuval Elani, Lorenzo Di Michele

**Affiliations:** † Department of Chemistry, Imperial College London, Molecular Sciences Research Hub, 82 Wood Lane, London W12 0BZ, U.K.; ‡ fabriCELL, Imperial College London, Molecular Sciences Research Hub, 82 Wood Lane, London W12 0BZ, U.K.; § Interdisciplinary Center for Nonlinear Phenomena and Complex Systems, Université Libre de Bruxelles (ULB), B-1050 Brussels, Belgium; ∥ Department of Chemical Engineering, Imperial College London, Imperial College Road, London SW7 2AZ, U.K.; ⊥ Department of Chemical Engineering and Biotechnology, University of Cambridge, Philippa Fawcett Drive, Cambridge CB3 0AS, U.K.

## Abstract

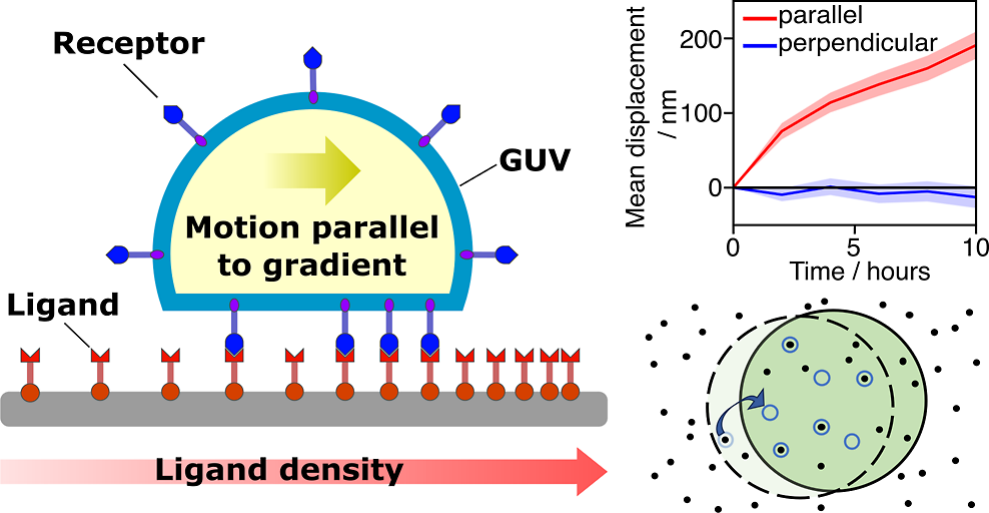

Multivalent adhesion between cell-membrane receptors and surface- or particle-anchored ligands underpins a range of active cellular processes, such as cell crawling and pathogen invasion. In these circumstances, motion is often caused by gradients in ligand density, which constitutes a simple example of haptotaxis. To unravel the biophysics of a potential passive mechanism for haptotaxis, we have designed an experimental model system in which multivalent lipid vesicles adhere to a substrate and migrate toward higher ligand densities. Adhesion occurs via vesicle-anchored receptors and substrate-anchored ligands, both consisting of synthetic DNA linkers that allow precise control over binding strength. Experimental data, rationalized through numerical and theoretical models, reveal that motion directionality is correlated to both binding strength and vesicle size. Besides providing insights into a potential mechanism for adhesive haptotaxis, our results highlight design rules applicable to the future development of biomimetic systems capable of directed motion.

## Introduction

Living cells interact with their environments via receptors embedded in their plasma membranes. These receptors can bind to specific ligand molecules, resulting in processes vital to biological function such as adhesion to external surfaces^1,2^ and the formation of pseudopodia involved in cell motility.^3^ Cell adhesion is often mediated by a large number of molecular bonds between cell-membrane receptors and surface ligands. These multivalent interactions can produce complex and useful emergent behaviors, such as binding superselectivity,^4,5^ due to the interplay between enthalpic and configurational effects.^6^ Numerous experimental and theoretical studies have been conducted to explore various aspects of multivalent interactions between particles, membranes and surfaces, including the strength and rate of adhesion;^7−11^ the growth, size and stability of the contact region between adhering objects;^12,13^ the self-assembly or fusion of colloidal particles;^14−20^ and receptor-mediated endocytosis.^21^

Cells and viruses adhering through multivalent interactions are known to perform directed motion. Examples include the diffusion of the Influenza A^22,23^ and Herpes^24^ viruses along the cell membrane prior to invasion; and the migration of mouse fibroblasts over cover-glasses coated with cellulose acetate and palladium.^25,26^

In many instances, the motion of adherent cells is directed along gradients of anchored ligand molecules, a type of motion known as haptotaxis, which has been observed in a variety of adherent cells, including mesenchymal stem cells, myoblasts, fibroblasts, leukocytes, cancer cells and Schwann cells.^27−37^ The mechanisms governing the haptotaxis of adherent cells are the subject of ongoing research. In some cases, the motion is considered to be driven by internal biochemical cascades capable of actively modifying the cell motility machinery to adapt their orientation along the gradient.^32^ Alternatively, adhesive haptotaxis could arise as a result of passive mechanical drift along the gradient due to a tug-of-war in the cell adherence zone, whereby areas with lower binding strength detach spontaneously in favor of areas with higher binding strength.^25,38^ Despite relevant studies on directed motion occurring in low valency systems,^39,40^ the biophysics of the passive drift of adherent cells along ligand gradients remains largely unexplored. In particular, the relative importance of factors such as binding strength and the size of the adhering cell or particle on the ability to perform haptotactic motion is yet to be clarified.

**Figure 1:**
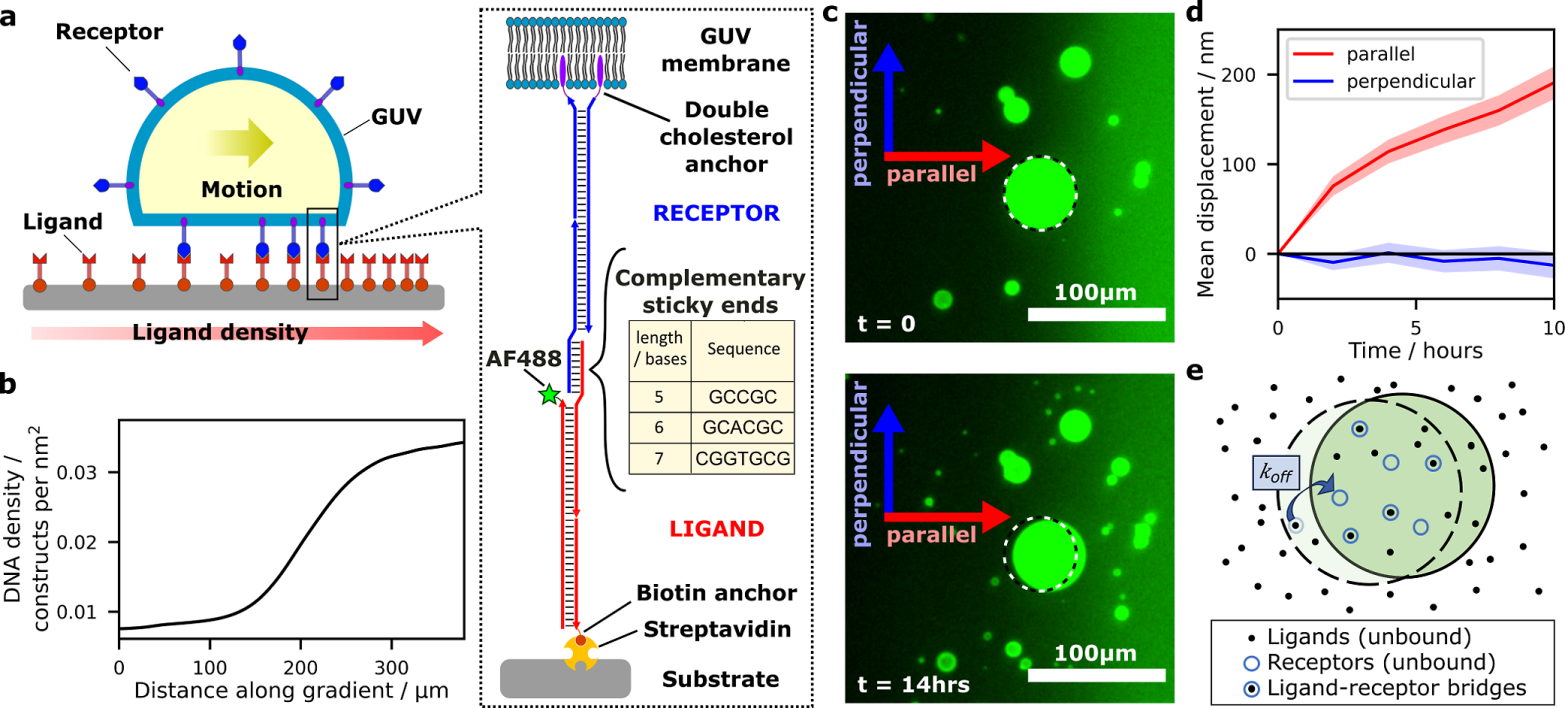
Overview of the experimental system. (a) Schematic of the experimental system, in which a giant unilamellar vesicle (GUV) functionalized with DNA “receptor” constructs interacts with a surface density gradient of complementary DNA “ligand” constructs attached to a substrate. Double-cholesterol anchors are used for anchoring the constructs to the GUVs, while biotin–streptavidin connections are used for the substrate. (b) Example of a typical ligand-density gradient profile from experiments. (c) Microscopy images showing the displacement of a GUV over 14 h; the original position of the vesicle is marked with a dashed circle. Axes indicating the directions parallel and perpendicular to the gradient are included. (d) Mean displacement over time of vesicles in systems with sticky end length *l* = 5 nt. Data for the mean displacement parallel and perpendicular to the ligand-density gradient are shown with the standard error of the mean shaded. Positive values of the displacement parallel to the gradient indicate motion toward higher ligand-density regions. (e) Schematic of the theoretical model, illustrating the motion of a GUV over a surface functionalized with ligands. The GUV is attached to the surface by receptor–ligand bridges, which constrain its position. Upon spontaneous unbinding of a bridge near its perimeter, the GUV can explore beyond its original constrained region.

Here, we address these questions using a combination of theory, numerical modeling, and experiments performed on a biomimetic model system. For experiments, we consider synthetic cellular mimics consisting of giant unilamellar vesicles (GUVs), interacting with a solid surface through multivalent adhesion. As schematized in Figure 1a, both the “receptors” on the GUVs and the “ligands” on the surface consist of synthetic DNA constructs interacting through selective base-pairing interactions that allow us to precisely modulate ligand–receptor affinity.^7,13,41^ A gradient in the surface density of the ligands is established, which causes the artificial cells to drift toward higher ligand concentrations thanks to the reversibility of the ligand–receptor interactions. We find that the vesicle drift velocity is approximately proportional to the unbinding rate of the ligand–receptor bridges, in agreement with theoretical considerations. We further explore the relationship between GUV size and motion, observing a positive correlation between vesicle size and drift velocity. Coarse-grained simulations based on the model developed in ref (42) produce consistent trends in vesicle motion when varying ligand–receptor affinity and vesicle size.

These results provide a deeper insight into the biophysics of a potential mechanism underlying adherent haptotactic motion, which could help to rationalize biological processes with relevance to immunity, host–pathogen interactions and tissue dynamics.^22,43,44^ Although the speed and range of motion of the GUVs in our experimental system are limited, our work provides knowledge to aid future designs of synthetic cells capable of moving or reorienting along surface-bound molecular gradients. Haptotactic cells could be used to generate a variety of phenomena such as coordinated motion in multicellular systems, or targeting and chasing of specific objects or chemical signals.^45−47^ Future designs with improved speed and range of haptotaxis, for instance aided by active mechanisms, could lead to the development of synthetic cellular solutions valuable in a variety of applications e.g. therapeutics and targeted drug delivery.^48−53^

## Results and Discussion

### Experimental System

A schematic of the experimental system is shown in Figure 1a. Electroformed GUVs prepared from 1,2-dioleoyl-*sn*-glycero-3-phosphocholine (DOPC), with typical diameters of 1–50 μm (see Figure S1), are functionalized with DNA constructs, here referred to as “receptors”. The receptors feature a double cholesterol anchor that irreversibly partitions within the lipid bilayer.^7,54^ As DOPC bilayers are fluid, receptors can freely diffuse laterally and redistribute across the surface of the GUV. The surface density of receptors is approximately 0.008 nm^–2^, estimated by calculating the ratio of DNA to DOPC molecules during the functionalization of GUVs, and assuming 100% efficiency of GUV synthesis, as well as an average area per DOPC headgroup of 72.5 Å. The receptors interact with a second set of DNA constructs, here referred to as “ligands”, which feature a biotin moiety for anchoring to a streptavidin-coated substrate. The receptor and ligand constructs feature complementary ssDNA sticky ends with lengths *l* ranging from five to ten nucleotides (nt), which can reversibly bind to each other. The constructs also feature rigid dsDNA spacers designed to control their spatial extent, and short poly-T domains to increase the flexibility and configurational freedom.^7^ The DNA sequences are listed in Table S1 with schematics of the constructs in Figure S2. We verified the correct assembly of the constructs and the binding of complementary receptor–ligand pairs using agarose gel electrophoresis (see Figures S3 and S4), while fluorescence microscopy was used to confirm attachment of the receptor constructs to the vesicle membranes (see Figure S5).

We set up a surface density gradient of ligands on the substrate, with density typically varying from 0.01 to 0.03 nm^–2^ over a distance of 100 μm (see Figure 1b). The methods for generating and characterizing the gradient are outlined in the Materials and Methods section, and images of typical ligand-density gradients are shown in Figure S6. Receptor-functionalized GUV are then deposited onto the gradient region where they adhere to the surface as sketched in Figure 1a. Note that the vesicles are randomly distributed over the surface. For sufficiently strong adhesion, the GUV take the shape of a truncated sphere, forming a flat adhesion patch visible in confocal cross sections (see Figure S7).^7,11,41^ Note that the electroformed vesicles naturally have some excess area without the need of an osmotic mismatch, allowing for deformation and the emergence of a flat adhesion patch.^13,41^

Time lapse epifluorescence microscopy videos are recorded, where both the GUV and the ligand-gradient can be visualized thanks to calcein dye loaded in the vesicles and Alexa Fluor 488 modifications on a subset of ligands. Example images in Figure 1c show a GUV migrating in the direction of the ligand-density gradient. Images are computationally segmented to identify vesicle trajectories (see Supporting Information Section 1). Projecting GUV displacement onto the directions parallel and perpendicular to the local gradient direction (see Supporting Information Section 1.3) allows us to quantify motion directionality, as exemplified in Figure 1d. Experiments are conducted at room temperature.

### Numerical Modeling

Coarse-grained simulations based on ref (42) are used to computationally characterize the multivalent haptotactic system. We map the vesicles onto 2D rigid disks of radius *R*, representing the perimeter of the flat adhesion patch, as shown in Figure 1e. In the following, we refer to the simulated objects as vesicles or disks, interchangeably. Thermal fluctuations in vesicle shape are neglected, and the disk-surface distance maintained constant. To keep simulations affordable, we use disks with diameter 2*R* = 0.2, 1, and 1.8 μm, an order of magnitude smaller than the GUVs used in experiments.

The surface is randomly decorated by ligands to generate a linear density profile with slope λ. The average surface density of the ligands at the starting location of the disks is set to ρ_L_ = 0.021 nm^–2^, while the average density of receptors on the vesicles is ρ_R_ = 0.008 nm^–2^, consistently with the nominal experimental values. The simulated gradient slope is set to λ = ρ_L_/10 μm^–1^, an order of magnitude higher than experimental gradients such that the steepness of the gradient relative to vesicle size is maintained. Note that further decreasing λ would rapidly make the system computationally untreatable, as for milder gradients the drifting component of the motion is overwhelmed by the stochastic one. Receptors on the vesicle can form bridges by binding free ligands within the projection of the disk onto the surface. The rate constants at which bridges form and break are given, respectively, by^7^
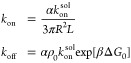
1where *k*_on_^sol^ is the hybridization rate of free oligonucleotides in solution; *L* is the length of the dsDNA spacer of the ligands and receptors; Δ*G*_0_ is the standard hybridization free energy of the sticky ends; ρ_0_ = 1 M is the standard concentration; and β = 1/(*k*_B_*T*), where *k*_B_ is the Boltzmann constant and *T* is the temperature. α is a nondimensional factor <1, accounting for the fact that the hybridization kinetics are expected to be slower as a result of the DNA constructs being tethered to surfaces. We set α = 0.1 as an estimate based loosely on observations made in ref (5). As simulated and theoretical drift velocities are proportional to α, changing this parameter affects the absolute values but not the trends generated by varying other system parameters. Vesicles are represented as hemispheres with a total surface area equal to 3π*R*^2^. Note that although the shape of adhered vesicles in experiments is likely to differ from a hemisphere, assuming a different shape for the calculation of *k*_on_ corresponds to a constant scaling factor on the drift velocity, and thus does not impact the trends reported in this paper. Accordingly, each free sticky end is taken as distributed within a layer of thickness equal to the ligand/receptor length (*L*) surrounding the vesicle’s surface. As the diffusion constants of DNA constructs anchored to lipid bilayers via cholesterol have been measured in the region of a few squared micrometers per second,^5,55^ we expect receptor redistribution to occur over time scales much shorter than experiments. We thus assume receptors to be uniformly distributed throughout this layer, with a resulting density equal to 1/(3π*R*^2^*L*). This density is used to calculate the binding rate *k*_on_ using standard reaction equations.^7^ By setting *k*_on_^sol^ = 10^6^ M^–1^ s^–1^ (an approximate estimate for short DNA oligomers^56^), *L* = 10 nm, *T* = 293.15 K (25 °C), and Δ*G*_0_ values estimated using the nearest neighbor thermodynamic model,^57−60^ we obtain *k*_off_ = 1.10, 0.174, and 0.0084 s^–1^ for sticky ends with *l* = 5, 6, and 7 nt, respectively.

In the model, we do not track the specific position of the receptors when free (i.e., not bound to a ligand), consistently with the observation that the receptors rearrange onto the bilayer much faster compared to the time scales of vesicle motion. At any point in time, the set of configurations available to the vesicle is limited by the formed bridges, as they are constrained to remaining inside the perimeter of the disk. Until the bonds rearrange, the disk can thus only rattle around the small subset of *n*_cb_ “constraining bridges” that can make direct contact with its perimeter (where ⟨*n*_cb_⟩ = 5^42^). Motion of the vesicle over larger distances can only emerge following reconfiguration of these constraining bridges, which thus limits motility.^42^ However, vesicle motion is not affected by nonconstraining bridges (i.e., bridges that do not make contact with the perimeter of the contact region), as the corresponding receptors can simply be dragged along the bilayer. Therefore, we cannot distinguish between sliding and tank-treading motion, in both the experiments and the model.

We simulate the system using a reaction-diffusion algorithm.^7,61^ At each simulation step, of duration Δ*t*, we iteratively employ the Gillespie algorithm^62^ to break existing ligand–receptor bridges or form new ones, and compute the time required for each reaction, until the total reaction time exceeds Δ*t*. Following the reaction step, we update the position of the disk by randomly (uniformly) selecting a new center of mass from all the possible locations compatible with the new configuration of bridges.^42^ The simulation code can be found online.^63^

**Figure 2:**
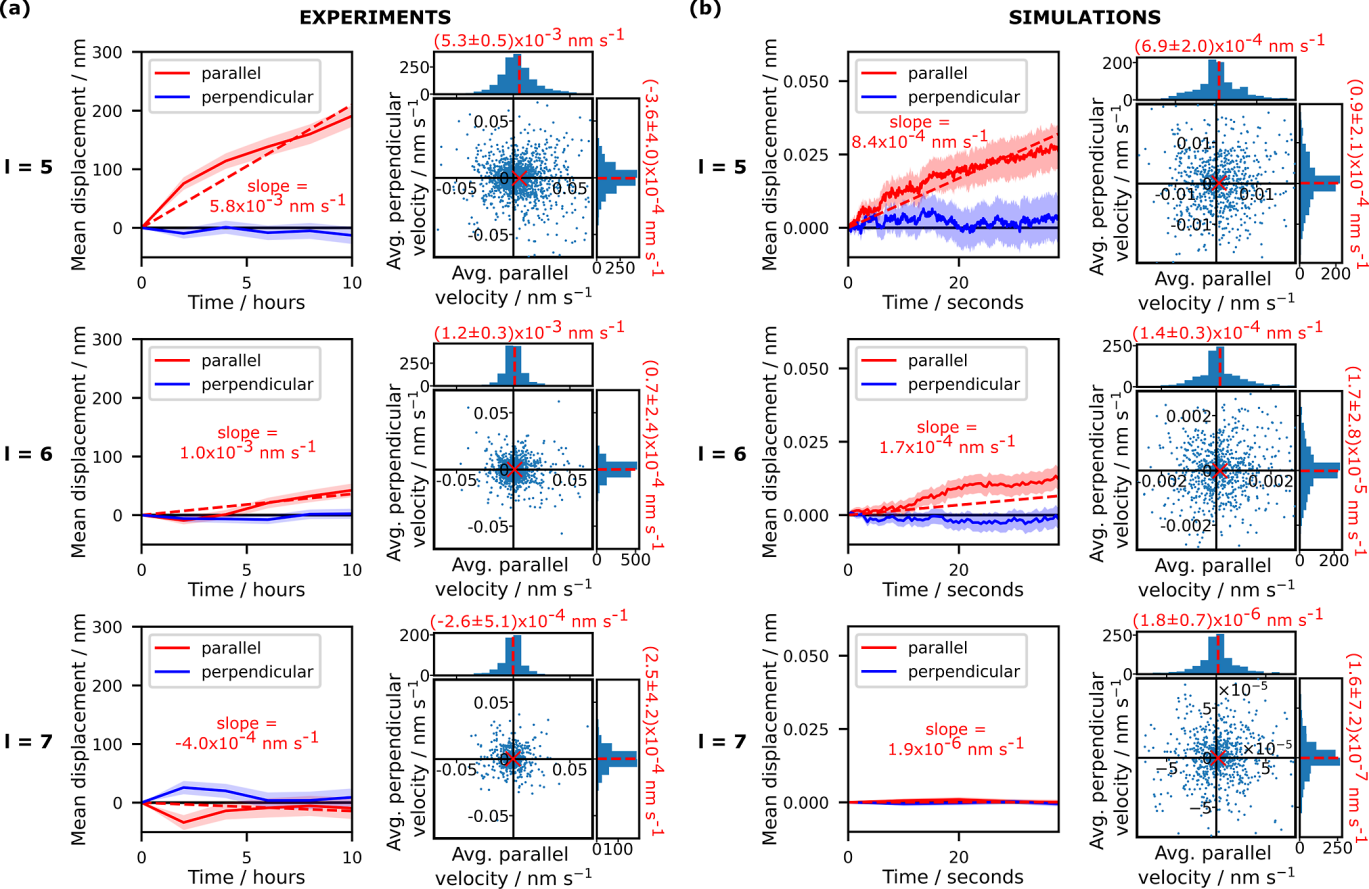
Drift velocity increases with decreasing sticky end length. Data illustrating the effect of sticky end length *l* on the motion of the GUVs along the ligand surface density gradient, for systems with *l* = 5–7 nt. Experimental data for vesicles of all sizes are shown in (a), while simulation data for 1 μm-diameter vesicles are shown in (b). Solid lines indicate the mean displacement of the vesicles parallel and perpendicular to the gradient over time, where positive values of velocity parallel to the gradient indicate motion toward regions of higher ligand density; the shaded regions indicate the standard error of the mean. Dashed lines indicate linear fits to the data for parallel motion, with the slope annotated. Simulated trajectories have been cropped to the same duration to enable visual comparison, while the straight lines have been fitted to the entire, uncropped trajectories (displayed in Figures S10 and S14). The scatter plots with marginal histograms show the average velocities of individual vesicles parallel and perpendicular to the gradient, calculated as total trajectory displacement divided by duration. The *y*-axes of the histograms indicate the number of vesicles in each bin. Red dashed lines and the red cross mark the mean of the distributions. Note that the axes have been cropped to aid visualization of the majority of data points. Graphs including all data points are shown in Figures S12 and S22.

**Figure 3:**
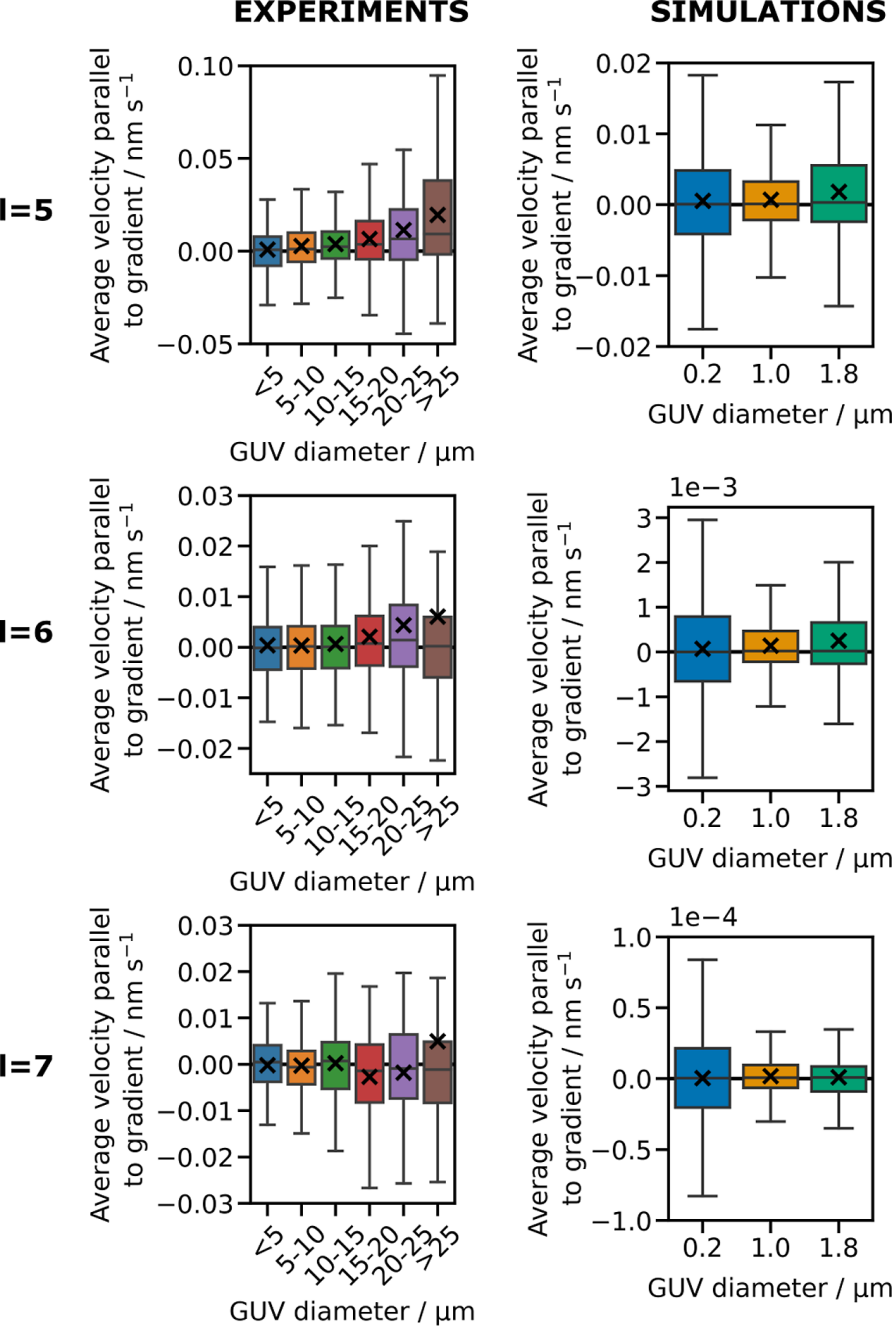
Drift velocity increases with increasing vesicle diameter. Box plots illustrating the relationship between GUV size and the average drift velocity of GUVs along the ligand density gradient, for sticky end lengths *l* = 5, 6, and 7 nt. The data show the distribution of average velocity along the gradient, which has been calculated as total displacement parallel to the gradient divided by trajectory duration. Positive values indicate motion of the vesicles toward regions of higher ligand surface density. For each box plot, the mean is marked with a cross. Note that outliers have been excluded for better visualization. Graphs with outliers included are presented in Figures S11 and S15.

The results of the simulation are shown in Figures 2 and 3 together with the experimental results.

### Weaker Binding Enhances Directional Motion

A key factor affecting the strength of multivalent binding and the corresponding vesicle mobility is the strength and (un)binding rates of individual receptor–ligand interactions. This factor can be tuned by varying the length of the complementary sticky ends; in our experiments and simulations we studied systems with sticky end lengths *l* = 5, 6, and 7 nt.

Experimentally, under all these conditions, the vesicles adhere to the substrate and form a stable, flat adhesion patch, as confirmed by confocal cross sections (see Figure S7). Previous experimental studies have shown that DNA constructs attached to fluid lipid bilayers via cholesterol anchors are mobile, and accumulate within the contact region between adhering bilayers driven by ligand–receptor affinity.^13,41^ Because the diffusion constants of the DNA constructs have been measured in the region of a few squared micrometers per second,^5,55^ we expect receptor redistribution in our system to occur over time scales much shorter than the duration of our experiments. When considering sticky ends in bulk solution at a nominal concentration corresponding to a uniform distribution of ligands and receptors within the contact region, NUPACK (nupack.org) produces estimates for their melting temperatures as ∼35, ∼41, and ∼49 °C for *l* = 5, 6, and 7, respectively.^64^ Despite the approximate nature of these estimates, which do not take into account the effects of sticky end tethering, it is still reasonable to expect a high probability of ligand–receptor dimerization at room temperature. Furthermore, using thermodynamic and geometric parameters along with nominal DNA concentrations, we predict that the number of bridges is saturated (and is limited by the density of ligands on the surface) for all DNA sequences employed in the paper (Figure S17). Note that saturation does not necessarily hamper drifting of the vesicle, as qualitatively explained by the theory. The reversibility of receptor–ligand binding allows the vesicle to still move, even if the receptor–ligand bridges are saturated.

As discussed in Section 1.4 of the Supporting Information, in all samples, we notice that a small proportion of vesicles (approximately 5–6%) remains much more static than the rest of the population, indicated by a significantly smaller diffusion coefficient. See Figure S8 for the distribution of diffusion coefficients, and Figure S9 for plots of mean squared displacement averaged over the “mobile” and “immobile” vesicle populations. We believe that this effectively immobile subpopulation could emerge due to a number of factors, such as sample impurities or surface defects being erroneously identified as vesicles, nonspecific adhesion or trapping of the vesicles on the substrate, or tracking inaccuracies. In the following data analysis the immobile population is excluded, although all plots in the main text have been replicated in the Supporting Information with the immobile population included for transparency (Figures S10–S15), showing no qualitative changes to the findings outlined below.

To study the speed and direction of the vesicle motion in both experiments and simulations, each trajectory was separated into components parallel and perpendicular to the local density gradient of DNA ligand constructs. Experimental and simulated data are collated in Figure 2a,b. We report the mean displacement, averaged over the population of sampled vesicles and projected along the directions parallel and perpendicular to the ligand-density gradient (Figure 2a,b, left). We stress that, different from the average displacement we study, the displacement of individual vesicles would be largely dominated by the stochastic component of the motion. We further show the two-dimensional distributions of average velocities, as computed from the initial and final positions of the vesicles in the trajectories (Figure 2a,b, right). From the average velocity distributions, we note that, in all cases, the motion is predominantly stochastic. However, while the projection of the average velocity onto the direction perpendicular to the gradient is centered around zero, i.e., shows no directional bias, the projection along the direction of the gradient has nonzero mean for *l* = 5 and 6 nt, both in experiments and simulations. The observed bias indicates motion toward denser regions of the ligand carpet, as expected. This result has been tested to show statistical significance, as discussed in Section 3.1 of the Supporting Information. For *l* = 7 nt, no clear directional bias is noted, in simulations or experiments. The width of the average velocity distributions also decreases with increasing *l*, indicating that vesicles with shorter sticky ends diffuse less, regardless of directionality, as shown in Figure S16. This relative decrease is more pronounced in simulations than it is in experiments, as expected given that experimental data are inevitably impacted by static localization errors.

Directional bias of the motion toward higher ligand densities is better visualized from the mean displacement data shown as a function of time (Figure 2a,b, left). In all cases, data on the motion perpendicular to the gradient average to zero (within standard error), while data on the parallel motion display a nonzero slope for *l* = 5 and 6 nt, with the latter being less pronounced. For *l* = 7 nt, mean displacement parallel to the gradient shows a negligible slope.

From the scatter plots in Figure 2 we calculate the average experimental drift velocities for the *l* = 5 and 6 nt systems to be 5.3 × 10^–3^ and 1.2 × 10^–3^ nm s^–1^ respectively. Over experimental time scales (i.e., 10 h), this equates to total displacements of 190 and 43 nm respectively, which are several orders of magnitude smaller than the typical vesicle diameter (≈10 μm; see Figure S1). However, we note that the distribution of vesicle displacements parallel to the gradient has a positive skew for both *l* = 5 and 6 nt, with maximum displacements along the gradient of 5.8 and 5.1 μm respectively, comparable with the average vesicle radius. For comparison, a freely diffusing vesicle of this size would travel ≈80 μm along the surface in this time scale (using the Stokes–Einstein equation to estimate the diffusion constant), which shows that the vesicle mobility is reduced by the multivalent adhesion.

To put the extent of the vesicle displacements into perspective, we consider the spacing between ligand–receptor bridges. In Figure S17 we see that the system is in a saturation regime where the ligand–receptor bridge density is limited by either the density of ligands or the density of receptors. This means that the bridge density is on the order of 0.01 nm^–2^, which corresponds to an interbridge spacing of 10 nm. The average vesicle displacement of ≈200 nm in the *l* = 5 nt system is 20 times higher than the interbridge spacing, while ≈10% of vesicles traveled further than 1 μm along the gradient, which is >100 times higher than the interbridge spacing. This indicates that the observed motion cannot simply be due to the rattling of vesicles around fixed bridging points but must require the formation and breakage of a large number of bridges.

Furthermore, we note that a typical vesicle with a diameter of 10 μm, located centrally in the gradient region of Figure 1b, spans ligand densities that only differ by ≈10% (from 0.019 to 0.021 nm^–2^). Therefore, during each formation of a new ligand–receptor bridge, it is only 10% more likely (at most) for the bridge to form on the side of the contact region facing toward higher ligand density, compared to the opposite side. This small difference in binding probability across the span of the vesicle suggests that a large number of bridge formation and breakage events are required for the directional vesicle drift along the gradient to be distinguishable from stochastic motion.

When comparing experiments with simulated trajectories, we note that the average drift velocities from experimental data for *l* = 5 and 6 nt are an order of magnitude higher than the simulated velocities (see Figure 2). This deviation could be due to the simulated vesicle diameters being an order of magnitude smaller than in experiments, although the simulated gradients have been adjusted to achieve the same gradient steepness relative to vesicle size. The effect of vesicle size on drift velocity is discussed later in this article. The difference in drift velocities between experiments and simulations could also be due to differences in the shape of the gradient profiles, or to unavoidable experimental artifacts such as small convective flows that contribute to errors in experimental estimates. Furthermore, calculations of the bridge formation and breakage rates (eq 1) for the simulations involve estimation of the correction factor α, which constitutes the biggest source of uncertainty in the simulated drift velocities. Note that if α is set to ≈1, the simulated drift velocities are then comparable to experimental drift velocities. This value of α would imply that the hybridization kinetics of tethered linkers are comparable to those of untethered linkers. Because of the above-mentioned factors, we argue that comparison of experimental and simulated drift velocities should be restricted to the trend observed in both when changing *l*, rather than absolute values.

Besides systems with sticky end lengths *l* = 5–7 nt, we also experimentally tested samples with shorter sticky end lengths (*l* = 3 and 4 nt), with the goal of further increasing vesicle mobility. However, we found that the binding strength was insufficient to keep the GUVs adhered to the surface, causing them to drift driven by convection or gravity (see Supporting Information Figure S18). No adhesion was observed regardless of vesicle size, indicating that even large GUVs interacting through a large number of receptors were unable to stick to the surface. It is possible that adhesion of larger vesicles was hindered by spurious convective flows or repulsive entropic effects linked to membrane fluctuation. The lack of adhesion in the *l* = 3 and 4 nt systems provides further evidence that the adhesion of vesicles in the *l* = 5–7 nt systems is due to the binding of complementary DNA constructs. We additionally designed constructs with longer sticky ends (*l* = 8–10 nt), which showed negligible drift consistent with the data in Figure 2 for *l* = 7 nt.

### Comparison of Experimental Drift Velocities with Theory

For a given number of ligands and receptors, the drift velocity is expected to be a function of the off rate (*k*_off_), the number of bridges (*n*_b_), and the diffusion constant of free (nonadhering) vesicles (*D*_free_). In systems featuring many bridges or low unbinding rates, the displacement dynamics of the vesicles is limited by the time scales of ligand/receptor reactions. This can be verified by comparing two characteristic time scales. The first is the typical time scale through which the translational configurational space available to the vesicles changes following a binding and unbinding of ligands/receptors, which can be estimated as τ_r_ = 1/(2*k*_off_*n*_cb_), with ⟨*n*_cb_⟩ ≈ 5.^42^ The second is the diffusion time scale needed by a vesicle to explore the available space: τ_d_ = π*R*^2^*n*_cb_/(*D*_free_*n*_b_^2^).

In conditions considered here, τ_r_ is at least 7 orders of magnitude bigger than τ_d_ (see Supporting Information Section 2.1), making the dynamics of our systems reaction-limited. Under these circumstances, we expect *v*_drift_ to be entirely determined by *k*_off_, resulting in the following scaling
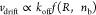
2where *f*(*R*, *n*_b_) is a general function on the variables *R* and *n*_b_.

For comparison, we derive an analytical prediction of the drift velocity. In our previous contribution, we predicted that a receptor-decorated disk on a uniformly ligand-decorated surface would diffuse with .^42^ By applying the fluctuation–dissipation theorem to this expression, we find (see Supporting Information Sections 2.2 and 2.4)
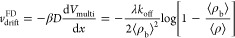
3where *V*_multi_ is the multivalent free-energy^7,65^ and ⟨ρ⟩ (⟨ρ_b_⟩) the density of (bound) ligands found at the center of the disk. The expression in eq 3 agrees with the relationship anticipated in eq 2. However, the theoretical prediction in eq 3 substantially overestimates drift velocities determined in both experiments and simulations, as shown in Figures S19 and S20. The discrepancies between eq 3 and simulations decrease at low values of *n*_b_. This discrepancy can be rationalized by noting that, for large *n*_b_ (and thus large drift forces), our systems may deviate from the linear response regime under which the fluctuation–dissipation theorem applies. This observation is in agreement with previous findings that coarse-grained dynamics in nonequilibrium conditions are not necessarily described by a simple Langevin equation.^66,67^ The relative magnitudes of the drift and diffusion components of the simulated trajectories are further discussed, and compared with theoretical predictions, in the Supporting Information, Section 2.3.

Although the theoretical prediction in eq 3 does not appear to be valid under experimentally relevant conditions, we still expect the drift velocity to follow the general scaling of eq 2. As previously mentioned, Figure S17 indicates that for *l* = 5, 6, and 7 nt, the system is in a saturation regime where the bridge density ⟨*n*_b_⟩ is limited by either the ligand density or the receptor density. It follows that the *R* and *n*_b_ dependencies in eq 2 should be unchanged when comparing experiments with different *l*, resulting in *v*_drift_ ∝ *k*_off_.

To verify the predicted proportionality, we compute the ratio between *v*_drift_ values for systems with different *l*, and compare it with the ratio between *k*_off_ values. Using the drift velocities extracted from the gradients of the straight line fits in Figure 2 (annotated to one decimal place on the plots), we calculate *v*_drift_^*l* = 5^/*v*_drift_^*l* = 6^ = 5.84 and 4.90 for experiments and simulations, respectively. Both these values are in good agreement with *k*_off_^*l* = 5^/*k*_off_^*l* = 6^ = 6.30, obtained using the values reported in the numerical modeling section of this article. For experiments, the small discrepancy possibly derives from using the same α and *k*_on_^sol^ for both sticky ends when computing *k*_off_ (eq 1), with both these parameters being, in principle, sequence dependent.^5,56^ For simulations, the deviation between *v*_drift_^*l* = 5^/*v*_drift_^*l* = 6^ and *k*_off_^*l* = 5^/*k*_off_^*l* = 6^ arises from statistical errors, given that the proportionality between drift velocity and *k*_off_^*l* = 5^ (eq 2) is enforced at the level of the simulation algorithm. While similar comparisons could not be carried out for the low-mobility system with *l* = 7 nt, the analysis for *l* = 5 and *l* = 6 nt supports a scenario where the drift velocity is dictated by unbinding rates, in alignment with the model leading to eq 2.

### Larger GUVs Exhibit Haptotaxis to a Greater Extent

We also experimentally observe that the size of the GUVs has an effect on the drift velocity along the gradient. When binning the experimental trajectories by vesicle diameter, we notice that larger vesicles travel on average a greater distance along the gradient, as shown in Figure 3 (left). This result has been found to be statistically significant, as discussed in Section 3.2 of the Supporting Information. The trend is most evident in the *l* = 5 nt system, where vesicles are the most mobile, but also present for the less mobile *l* = 6 nt system. Simulated trajectories for vesicles of varying diameters confirm the experimental trends for *l* = 5 and 6 nt (see Figure 3, right). In both the experiments and simulations, we found that the average vesicle drift velocity is typically greater than the median, due to the velocity distribution having a positive skew. We do not observe a clear relationship between vesicle size and motion along the gradient for *l* = 7 nt, in neither experiments nor simulations, likely due to any signal being drowned by noise in this low-mobility system. Regardless of size, we observe that, on average, vesicles drift a negligible distance along the direction perpendicular to the gradient, as shown in Figures S13 and S23 and consistent with the data in Figure 2.

One possible explanation for the relationship between vesicle size and drift velocity in the mobile systems (*l* = 5 and 6 nt) is that larger vesicles cover a greater extent of the ligand density gradient, corresponding to a greater change in binding affinity across the width of the vesicle. Following from the discussion above, for a vesicle located centrally on the gradient depicted in Figure 1b, the ligand density varies by ∼ 5% across a 5 μm-diameter contact region, and by ∼20% across a 20 μm-diameter contact region. This means that the larger vesicle would have a higher rate of bridge formation on the side of the contact region facing up the gradient compared with the smaller vesicle, resulting in a greater drift velocity. This effect has also been discussed in ref (68), where the motility of influenza virus particles adhered to surface-bound molecular density gradients was studied. In this case, no directional bias was observed in the virus motion, which the authors ascribed to the small size of the virus particles relative to the steepness of the gradient in their system.

We also note that while larger vesicles can more effectively sense gradients, they are also less mobile due to the larger number of bridges.^42^ These two competing factors perfectly compensate each other in the theoretical prediction reported in eq 3, where *v*_drift_^FD^ is not dependent on *R*. Evidence of a size-dependent drift velocity in both experiments and simulations further highlights the limitations of the theoretical approach leading to eq 3 in the strong-binding regime relevant to our system.

Given our understanding of the impact of vesicle size relative to the gradient steepness on the drift velocity, we then argue that varying vesicle size for a given gradient is the equivalent of varying gradient steepness for a given vesicle size. Therefore, our experimental results indicate that increasing the gradient steepness would result in increased drift velocities. Although our experimental method of generating ligand-density gradients does not allow for tuning of the gradient profile, we did explore the effect of varying the gradient steepness on drift velocity in our simulations, as reported in Figure S20. As expected we observe that the drift velocity of vesicles along the gradient increases with gradient steepness. Meanwhile, we observe negligible drift perpendicular to the gradient regardless of gradient steepness, as illustrated in Figure S21.

## Conclusions

In summary, through experiments, theory and simulations, we studied the directional motion of receptor-decorated lipid vesicles adhering to a ligand-decorated surface, in the presence of a gradient in ligand density—a model system for haptotactic crawling. Experimentally, both receptors and ligands consisted of synthetic DNA constructs, the former connected to giant unilamellar lipid vesicles and the latter to a solid surface. Coarse-grained simulations relied on a multiscale approach first reported in ref (42), which accurately describes the binding and unbinding dynamics of ligands and receptors through a Gillespie algorithm.

Both simulations and experiments showed directional motion of the vesicles toward ligand-dense regions of the surface. The magnitude of the directional drift was observed to decrease with increasing ligand–receptor affinity, which could be easily controlled by changing the length of the single-stranded DNA sticky ends through which our constructs interact.

With theoretical arguments we demonstrated that, in the regime relevant to our system, directional motion is limited by the time scales of the ligand–receptor reactions. The corresponding relationship between drift velocity and ligand–receptor unbinding rates was confirmed by both experiments and simulations.

We also observed a positive correlation between vesicle size and drift velocity in both experiments and simulations. This trend, more noticeable in more mobile systems, is explained by the ability of larger vesicles to probe a greater extent of the ligand density gradient, thus generating greater spatial asymmetry in the ligand–receptor binding probabilities.

Our findings offer quantitative insights on the mechanisms underpinning directional motion in multivalent systems, highly relevant to a variety of biological processes involving membrane interactions. Examples include immune cells adhesion,^43,69^ viral invasion,^22−24^ and tissue dynamics.^22,43,44^ Although the drift velocities of vesicles reported in this article are small in comparison with the speeds of crawling mammalian cells, the passive haptotactic drift mechanism could have relevance to the reorientation of cells. For example, malaria cells release adhesin from the apical region of the parasite, producing a transient gradient in binding energy that results in reorientation of the cell.^70^ In future studies, it would be interesting to explore parameter spaces such as temperature, buffer conditions and gradient steepness, as well as working in more weakly binding regimes (i.e., by reducing sticky end length or decreasing DNA density); this may require alteration of the experimental setup to reduce convection effects. It would be furthermore valuable to enhance the binding/unbinding dynamics of the system through enzymatic processes, which may greatly enhance drifting velocities.^71,72^

Our models and experimental implementation will also support efforts to engineer directed motion in synthetic cellular systems. Mobile synthetic cells capable of performing simple haptotaxis would indeed be valuable for a vast range of applications, such as smart drug delivery systems that can target and release drugs at specific locations in the body;^48−53^ vesicle-based biosensors or biomedical imaging systems that signal the presence of specific molecules or conditions;^73−75^ or tissue engineering applications where gradients of molecules or growth factors are used to direct cell migration and tissue formation.^76−78^

## Materials and Methods

### Experimental Materials

#### DNA Oligonucleotides

Sequences of the DNA oligonucleotides were designed using the NUPACK design tool.^64^ DNA strands with 5′-cholesteryl modifications were purchased from Eurogentec (Liege, Belgium) with high performance liquid chromatography (HPLC) purification. All other DNA strands were purchased from Integrated DNA Technologies (Coralville, Iowa, United States). Modified strands were purified via HPLC, and unmodified strands were purified by standard desalting.

#### Buffers

NaCl (BioUltra, >99.5%) and 100× Tris EDTA (TE) buffer were purchased from Sigma-Aldrich (Gillingham, UK). 10× Tris Borate ETDA (TBE) buffer was purchased from ThermoFisher Scientific (UK). All buffer solutions were diluted with the appropriate amount of Milli-Q water, and were filtered through 0.22 μm pore poly(ether sulfone) filters (Millex) prior to use.

#### Gel Electrophoresis

Agarose was purchased from Sigma-Aldrich (Gillingham, UK). Ultra Low Range DNA Ladder and 6× TrackIt Cyan/Yellow Loading Buffer were purchased from ThermoFisher Scientific (UK). 10,000× SYBR Safe DNA gel stain was purchased from APExBIO Technology LLC (Houston, Texas, USA).

#### Vesicle Generation

d-(+)-Glucose (>99.5%), sucrose, chloroform and indium tin oxide coated glass slides (surface resistivity 15–25 Ω sq^–1^) were purchased from Sigma-Aldrich (Gillingham, UK). > 99% 1,2-Dioleoyl-*sn*-glycero-3-phophocholine (DOPC) and 1,2-dioleoyl-*sn*-glycero-3-phosphoethanolamine-*N*- (7-nitro-2–1,3-benzoxadiazol-4-yl) (ammonium salt) (NBD-PE) was purchased from Avanti Polar Lipids Inc. (Alabaster, Alabama, USA).

#### Well Plates and Adhesive Covers

Streptavidin-coated high capacity 96-well strip plates were purchased from Sigma-Aldrich (Gillingham, UK). Adhesive clear foils for 96-well plates were purchased from Sarstedt Ltd. (Leicestershire, UK).

### Experimental Methods

#### DNA Reconstitution

The DNA strands were shipped lyophilized and were reconstituted to a concentration of approximately 100 μM in 1× TE buffer (1 mM EDTA, 10 mM Tris, pH 8.0). The concentration of the reconstituted DNA was determined via UV–vis spectrophotometry on a Thermo Scientific NanoDrop One, by measuring the ratio of absorbance at 260 nm to the sequence-specific extinction coefficient of the DNA. Stock solutions of DNA were then stored at −20 °C.

#### DNA Hybridization

Assembly of the multistranded receptor and ligand constructs was facilitated by thermal annealing. For each construct, the constituent strands were diluted to 2 μM and combined in TE buffer containing 100 mM NaCl. The strands were first heated to 90 °C for 5 min to ensure melting of all DNA duplexes, and then cooled to 20 °C at a rate of −0.5 °C min^–1^, on a Bio Rad C1000 Thermal Cycler. The sizes of the annealed DNA constructs were evaluated via agarose gel electrophoresis to verify correct folding, as reported in Figures S3 and S4.

#### Agarose Gel Electrophoresis

3% (w/v) agarose gels were casted in 1× TBE buffer with 1× SYBR Safe DNA gel stain. The 10 μL wells were loaded with approximately 300 ng DNA samples with loading dye; the outer two wells were loaded with DNA ladders, to enable comparison with the DNA samples. The gels were run for 90 min at 120 V (electric field strength of 6 V cm^–1^) and then imaged using a Syngene Dyversity 4 gel imager.

#### Microscopy

Epifluorescence microscopy imaging was performed on a Nikon Eclipse Ti2-E inverted microscope using a Nikon CFI Plan Apochromat Lambda D 10× dry objective (NA 0.45). Confocal microscopy imaging was performed on a Leica TCS SP5 Confocal microscope using a HCX PL APO CS 63.0× oil-immersion objective (NA 1.40).

#### Preparing Vesicles

The GUVs were produced via electroformation, a straightforward and commonly used method for vesicle generation.^79^ Lipid solutions consisting of DOPC with 1 mol % fluorescent lipid NBD-PE were prepared by dissolving the appropriate quantities of lipids in chloroform to yield a 1 mg mL^–1^ solution. 30 μL of this solution was spread evenly on an Indium Tin Oxide (ITO) slide and vacuum-desiccated for 30 min to remove residual chloroform, resulting in a lipid film. A 5 mm-thick polydimethylsiloxane (PDMS) spacer with a central cut-out was sandwiched between this film and another ITO slide to create a chamber, and the chamber was filled with a solution consisting of 300 mM sucrose and 50 μM calcein in water. A function generator (Aim-TTi, TG315, Huntingdon, UK) was used to apply an alternating electric field at a peak-to-peak voltage of 1.5 V was applied across the ITO slides at 10 Hz for 2 h, followed by one further hour at 2 Hz. During this time, the chamber was left in a 60 °C oven to ensure that the lipid was in the fluid phase. Finally, the chamber was opened and the resulting vesicles were collected. The vesicle sample was inspected via epifluorescence microscopy, by exciting both the NBD-PE fluorophore and the encapsulated calcein. Note that the calcein signal overwhelms the signal from the NBD-PE; the latter was included to aid visualization of the vesicles in the case of any calcein leakage. Ease of imaging can be improved by mixing the vesicle sample with a solution of 300 mM glucose in Milli-Q water, such as in a 1:10 ratio of vesicles to glucose solution. This introduces a density difference which causes the vesicles to settle at the bottom of the viewing chamber, and a refractive index contrast to enable viewing in brightfield. The size distributions of vesicles in our experiments are shown in Figure S5.

#### Vesicle Functionalization

GUVs were functionalized by incubation with cholesterolised DNA constructs. A sample was prepared with 20 v/v % 2 μM DNA constructs in 100 mM NaCl TE buffer, 11 v/v % GUVs (prepared as detailed in the previous paragraph) in 300 mM glucose solution, and 69 v/v % “correction buffer”. This correction buffer consists of 116 mM NaCl, 78.2 mM glucose, and 1.16× TE buffer, and was included to match the osmolarity of the interior and exterior of the GUVs and thus to prevent osmotic-shock-induced rupture. The sample was left to incubate on a roller mixer overnight. After the functionalization process, the vesicle sample was washed to minimize the amount of DNA constructs remaining in solution that have not been incorporated into the lipid membranes. This was achieved by leaving the sample to stand for at least 15 min to allow the vesicles to settle, and then replacing approximately 90% of the eluent with an iso-osmolar solution consisting of 100 mM NaCl and 87 mM glucose in TE buffer. The washing process was carried out at least three times. To check that the vesicles had been successfully functionalized and the lipid membranes were saturated with DNA constructs, the vesicles were prepared with fluorescently tagged lipids and DNA constructs, and imaged via epifluorescence and confocal microscopy. The estimated density of DNA constructs in the lipid membrane is approximately 0.008 nm^–2^.

#### Ligand-Density Gradient Generation and Characterization

A 0.5 μL droplet of 2 μM biotinylated DNA “ligand” constructs in buffer solution (100 mM NaCl and 1 × TE) was deposited on the substrate, a well plate precoated with streptavidin. This was left to incubate for 5 min to allow the DNA constructs to bind to the substrate. Excess DNA was then washed away by quickly rinsing the substrate with buffer solution (100 mM NaCl and 1 × TE). This left a coating of DNA in the circular region that had been covered by the droplet, with a diameter of ≈3.5 mm and a ligand density of ≈0.033 nm^–2^. Due to the fast rate of binding between streptavidin and biotin, a small amount of excess DNA bound to the surrounding region during the washing process; the surface density of DNA in this region was measured to be 0.005–0.01 nm^–2^ on average. At the perimeter of the circular maximally coated region, there was a steep surface density gradient spanning a distance of approximately 300 μm. The ligand constructs were tagged with Alexa Fluor 488, enabling the ligand coverage to be imaged and characterized by excitation of the fluorophore.

The ligand density in the maximum-density region of the wells was estimated by applying a 0.5 μL droplet of 2 μM ligand solution (with the Alexa Fluor 488 modification) to the well for 5 min, like in the gradient generation procedure. The supernatant was then diluted by a factor of 200 by adding 99.5 μL of buffer solution (100 mM NaCl and 1 × TE) and mixing with a pipet. 80 μL of the diluted supernatant was extracted, and its fluorescence was recorded on a BMG LABTECH CLARIOstar Plus microplate reader. To estimate the quantity of ligand constructs that had been deposited on the substrate, the fluorescence of the diluted supernatant was compared with the fluorescence of control samples i.e. 80 μL ligand solutions with known concentrations (0, 0.1, 1, 5, and 10 nM) and the same buffer conditions (100 mM NaCl and 1 × TE). Note that all samples (including the sample with unknown ligand concentration) were repeated four times to improve measurement accuracy; background signal was removed from the fluorescence measurements by subtracting the average fluorescence intensity of the control samples with 0 nM ligand concentration. A straight line was fitted to the fluorescence of the control samples, to determine a calibration curve between ligand concentration and fluorescence signal (see Figure S24). From this, it was deduced that the ligand concentration of the supernatant of the droplet applied to the well (diluted by a factor of 200) was 3.0 nM, which corresponds to a loss of 70% of ligands from the droplet solution. Assuming that this loss is entirely due to binding of ligands to the well within the droplet region, and an approximate diameter of the droplet region of 4 mm, this corresponds to an estimated density of 0.33 nm^–2^ of ligands bound to the well within the droplet region.

To characterize the ligand-density gradient profiles in experiments, the ligand coverage was imaged with epifluorescence microscopy. Due to nonuniform illumination of the sample, the fluorescence images were normalized by a control image taken within a nonfunctionalized well containing calcein solution (see Figure S6). The ligand density was then estimated by interpolating between the background fluorescence signal (recorded from a nonfunctionalized well) and the average fluorescence signal of a maximum ligand-density region of the well. The local direction and magnitude of the ligand density gradient were calculated at each point in the image, as depicted in Figure S25.

#### Recording Vesicle Motion

After generating the ligand-density gradients, the receptor-coated vesicles were pipetted into the wells, and the well plate was covered with a transparent adhesive foil to minimize evaporation and evaporation-driven flows in the sample. The sample was then left to sit for 1 h to allow most of the vesicles to sink to the bottom of the well and adhere. A time lapse recording was then taken of the vesicles on the substrate, over a duration of 10–15 h. For the purposes of aggregating data across experiments and presenting results in the main text, experimental trajectory data were cropped to the duration of the shortest experiment (10 h). Note that all experiments were conducted at room temperature.

#### Vesicle Tracking

The time lapse recordings were initially stabilized using the *Image Stabilizer* plugin on ImageJ^80^ to remove any unwanted effects of stage drift or inaccuracies in stage positioning. The image data were then loaded into Python, and processed to remove noise and prepare the images for the tracking algorithm. Vesicles were located in each frame of the time lapse recordings using a template-matching method. Particle trajectories were then identified by linking particle positions between successive time frames; this was achieved using the Python module *Laptrack*.^81,82^ More details on data processing and the vesicle locating and tracking algorithms can be found in Section 1 of the Supporting Information.

## Supporting Information Available

Details of data processing methods for vesicle-tracking; additional details of numerical methods; test results for statistical significance of vesicle drift; table of DNA strand sequences; supplementary figures illustrating details of the experimental setup, further analysis of vesicle drift in experiments and simulations, and the theoretical prediction of receptor–ligand bridge density (PDF)
