# Supplementary material for: Haptotactic Motion of Multivalent Vesicles Along Ligand-Density Gradients
Source: Langmuir. 2025 Apr 29;41(18):11474–85. doi: 10.1021/acs.langmuir.5c00494 (PMC12080341; doi:10.1021/acs.langmuir.5c00494)
Supplement: Supplementary file 1 — la5c00494_si_001.pdf [file la5c00494_si_001.pdf]

Supporting Information: Haptotactic motion of multivalent vesicles along ligand-density gradients

Hannah Sleath,[†,‡] Bortolo M. Mognetti,[*,¶] Yuval Elani,[*,§,‡] and Lorenzo Di Michele[*,‖,†,‡]

†*Department of Chemistry, Imperial College London, Molecular Sciences Research Hub, 82 Wood Lane, London W12 0BZ, United Kingdom*

‡*fabriCELL, Imperial College London, Molecular Sciences Research Hub, 82 Wood Lane, London W12 0BZ, United Kingdom*

¶*Interdisciplinary Center for Nonlinear Phenomena and Complex Systems, Université Libre de Bruxelles (ULB), B-1050 Brussels, Belgium*

§*Department of Chemical Engineering, Imperial College London, Imperial College Road, London SW7 2AZ, United Kingdom*

‖*Department of Chemical Engineering and Biotechnology, University of Cambridge, Philippa Fawcett Drive, Cambridge CB3 0AS, United Kingdom*

E-mail: bortolo.matteo.mognetti@ulb.be; y.elani@imperial.ac.uk; ld389@cam.ac.uk

1 Supplementary data processing methods

1.1 Pre-processing vesicle time lapse data

The time lapse recordings of vesicles over DNA surface density gradients were originally stabilised using the *Image Stabilizer* plugin on ImageJ[1] to remove any unwanted effects of stage drift or inaccuracies in stage positioning. The image data were then loaded into Python, and processed to remove noise and prepare the images for the tracking algorithm. The stages of image processing were as follows:

- A top-hat transform was applied to the images, to flatten the non-uniform fluorescence signal from the DNA coating on the substrate.
- Gaussian smoothing was applied to the images, to remove some fluorescence measurement noise.
- The images were binarised, to discern the fluorescent vesicles from the background signal.
- Morphological opening operations were applied to the images, to remove noise (*i.e.* bright specks) from the dark background signal.
- Morphological closing operations were applied to the images, to remove noise (*i.e.* dark specks) from the bright vesicles.

1.2 Identifying vesicle trajectories

Vesicles were located in each frame of the time lapse recordings using a template-matching method. The stages of this method were as follows:

- As the vesicle samples are polydisperse, direct application of a template matching method to search for bright circular objects in the binarised images did not perform

sufficiently well. For instance, the algorithm would sometimes incorrectly identify a larger vesicle as comprising of multiple smaller vesicles. Therefore, in order to achieve size-invariant identification of vesicles in the images, the distance transform was used. The distance transform was applied to the binarised image data, labelling each pixel with the distance to the nearest dark pixel.

- The distance transform of the vesicle template (also referred to as the kernel) was generated. This template provides the basic shape which the template-matching algorithm searches for in the images.

- A template-matching algorithm (the *matchTemplate* functionality of the Python OpenCV module) was applied to the image data, identifying regions in the images which closely match the template (*i.e.* bright disks in the original image). This generated an array of values indicating the similarity of each region of the image to the template.

- This array of values was then binarised, to select regions of the image with the highest similarity to the template, which should correspond to the vesicles in the image.

- The position and value of the local maxima within each non-contiguous region of high similarity were determined, to provide an initial approximation of the vesicles' positions and radii.

- To improve the position and radius estimates to sub-pixel accuracy, a function-fitting approach was employed. We defined a function that outputted the distance transform of a bright disk, depending on the position and size of the disk. We then fitted this function to the local regions of the image in which a vesicle had been approximately located. The fitted model parameters provide a new, more accurate estimate of the vesicle positions.

- To try to reduce any remaining noise or inaccuracies, we removed vesicles with an estimated radius of less than one pixel and vesicles overlapping with the edges of the

image from the data.

- The data was filtered to only include vesicles in the relevant regions of the images *i.e.* in the uniform-density regions or the density-gradient regions. For the former, we manually cropped the data to regions of uniform density; for the latter, we applied a script that identified regions of the image in which there was a steep gradient of DNA density, and then manually cropped out any remaining data in regions that had been incorrectly selected.

Vesicle trajectories were then identified by linking vesicle positions between successive time frames; this was achieved using the Python module *Laptrack*.[2,3] This module employs a linear-assignment-problem-based approach to identifying vesicle trajectories.

1.3 Identifying components of vesicle motion parallel and perpendicular to gradient

The fluorescence image of the DNA coating on the substrate (prior to addition of the vesicles) was processed as follows:

- Gaussian smoothing was applied to the image to reduce noise in the fluorescence signal.
- To account for any uneven illumination or camera sensitivity across the frame of view, the image was normalised by a control image of a uniform fluorescence signal. This control image was attained by imaging a well filled with calcein solution.

The vector gradient (also referred to as the directional derivative) of the fluorescence image of the DNA-coated substrate was computed. The resulting vector field describes the magnitude and direction of the density gradient at each point in the image. A plot of a gradient vector field superimposed on the corresponding fluorescence image of a substrate coated with ligands is shown in Fig. S25. Finally, we calculated the components of the vesicle velocities parallel and perpendicular to the direction of the gradient. This was achieved by calculating the

vector dot and cross products (respectively) of the vesicle velocities with the unit vectors describing the direction of the gradient at each vesicle position.

1.4 Mean squared displacement analysis and filtering of vesicle data

We employed mean squared displacement (MSD) analysis to study vesicle motion within our experimental data. For each vesicle trajectory, we calculated the MSD for a given lag time τ by slicing the trajectory into sections of duration τ and averaging the square displacement over these sections, as follows:

$$MSD(\tau) = \frac{1}{\lfloor \frac{T}{\tau} \rfloor} \sum_{n=0}^{\lfloor \frac{T}{\tau} \rfloor - 1} (\vec{r}_{(n+1)\tau} - \vec{r}_{n\tau})^2 \tag{1}$$

where T is the total recorded duration of the trajectory, and $\vec{r}_t$ is the position of the vesicle at time t.

We then plotted the MSD as a function of the lag time, to identify the type of motion. In general, particles undergoing Brownian diffusion exhibit a linear relationship between MSD and lag time, while particles travelling with directed motion exhibit a quadratic relationship.[4,5]

To gain an initial insight into our experimental data, we used a least-squares approach to fit straight lines to the plots of MSD versus lag time. The straight lines were only fitted to values of MSD that had been averaged over at least two trajectory slices, and non-negative constraints were enforced on the intercept and slope of the fitted lines, as negative values would be non-physical and indicate tracking errors. We then extracted rough estimates of the vesicle diffusion constants from the slope of the line. Upon plotting histograms of the estimated diffusion constants, we observed bimodal distributions as seen in Figure S8, with two distinct peaks corresponding to a “faster" and “slower" vesicle population. For each of the $l = 5$, 6 and 7 nt sticky end systems, approximately 5–6% of the vesicles appear to belong to the “slower" population.

To understand the underlying factor behind the bimodal distribution, we inspected the MSD plots as a function of lag time for vesicles in the “slower" and “faster" populations of the bimodal distribution. We found that for the majority of the vesicles in the “slower" population, the MSD appeared to have a decreasing trend with increasing lag time. Due to the non-negative constraints on the intercept and slope of the linear fits, the fitting algorithm assigned negligible, near-zero values to the fitted parameters, resulting in an apparent secondary population of slower-moving vesicles.

We then studied the trajectories of the vesicles with an apparent negative relationship between MSD and lag time, by comparing the algorithmically tracked trajectories with the imaged vesicle motion by eye. In the majority of these cases it appeared that inaccuracies and noise in the particle tracking had resulted in the negative relationship between MSD and lag time.

We also observed by eye that a small minority of vesicles in our experiments appear to be immobile. This could in part be due to non-uniformity in DNA density on the substrate and vesicle surfaces resulting in vesicles being trapped in high density regions. However, we still observed a small proportion of immobile vesicles in systems where no DNA ligands or receptors were present. We believe that these immobile vesicles may be become pinned to surface defects, or trapped by strong non-specific adhesion. Bimodal distributions of particle motility with an immobile population have also been observed in other particle motility studies.[6]

Finally, we note that erroneous tracking of sample impurities or surface defects as vesicles could contribute to the apparent population of immobile vesicles.

We chose to discard the apparent “slow" population of vesicles from the experimental data from further data analysis, by excluding vesicles with a diffusion constant estimated to be less than 10^{-8} $\mu m^2 s^{-2}$. This would reduce the number of inaccurate trajectories included in our data analysis, and also the number of vesicles that are immobile for non-DNA reasons. However, for transparency, we present our results pre- and post-filtering in the SI.

2 Supplementary numerical methods

2.1 Evaluation of timescales relevant to vesicle dynamics

2.1.1 Reconfiguration timescale of constraining bridges

The translational configurational space available to the vesicle depends on a set of constraining bridges near the perimeter of the contact region. The timescale of the reconfiguration of these constraining bridges can be estimated as $\tau_{\rm r} = 1/(2k_{\rm off}n_{\rm cb})$, where $k_{\rm off}$ is the rate at which bridges break, and with the average number of constraining bridges $\langle n_{\rm cb}\rangle \approx 5$.[7] From Eq. 1 in the main text we calculated $k_{\rm off} = 1.10\,{\rm s}^{-1}$, $0.174\,{\rm s}^{-1}$, and $0.0084\,{\rm s}^{-1}$ for sticky ends with $l = 5$, 6, and 7 nt respectively, under experimental conditions. Using these values, we estimate the timescales of constraining bridge reconfiguration to be 0.091 s, 0.57 s, and 11.9 s for sticky ends with $l = 5$, 6, and 7 nt respectively.

2.1.2 Diffusion timescale

The diffusion timescale needed by a vesicle to explore the available configurational space can be estimated as $\tau_{\rm d} = \pi R^2 n_{\rm cb}/(D_{\rm free}n_{\rm b}^2)$, where $D_{\rm free}$ is the diffusion constant of free (non-adhering) vesicles. The Stokes-Einstein equation can be used to estimate the diffusion constant of a 5 μm-radius vesicle as $0.049\,\mu{\rm m}^2{\rm s}^-1$. In Fig. S17 we note that the bridge density is comparable to the ligand and receptor densities, *i.e.* on the order of $0.01\,{\rm nm}^{-2}$. Using these values, we estimate the timescale for the vesicle to explore the available configurational space via diffusion to be $1.30{\times}10^{-8}$ s.

2.2 Theoretical prediction of vesicle drifting velocity

We postulate that the dynamics of the vesicles' center of mass ($x_{\rm CM}$) is described by an overdamped Langevin equation

$$\frac{\partial x_{\rm CM}}{\partial t} = -\beta D(x_{\rm CM})\frac{\partial F(x_{\rm CM})}{\partial x_{\rm CM}} + f_1(x_{\rm CM}) + \sqrt{2D(x_{\rm CM})}\eta(t) \quad (2)$$

with $\beta = 1/(k_B T)$. $F(x_{\rm CM})$ is the multivalent free-energy of the vesicle at a given $x_{\rm CM}$,[8] $\eta(t)$ a Gaussian white noise ($\langle\eta(t)\rangle = 0$, $\langle\eta(t)\eta(t')\rangle = \delta(t-t')$), and $D(x_{\rm CM})$ the position-dependent diffusion constant.[7] To fully determine the solutions of Eq. 2, one needs to specify a prescription to interpret the stochastic term of this equation in which the noise source multiplies a non-constant function. Such a prescription identifies the value of the following integral

$$\int_t^{t+\Delta t} {\rm d}t' g(x(t'))\eta(t') = g(\gamma x(t+\Delta t) + (1-\gamma)x(t)) \int_t^{t+\Delta t} {\rm d}t' \eta(t') \tag{3}$$

where γ is a parameter with $\gamma \in [0, 1]$. For instance, $\gamma = 0$, $1/2$, and 1 when considering, respectively, the Ito, Stratonovich, and isothermal convention. Ref.[9] has shown the necessity of considering an extra drifting term in the Langevin equation ($f_1(x_{\rm CM})$ in Eq. 2) to ensure recovery of the expected equilibrium state, $P(x_{\rm CM}) \sim \exp[-\beta F(x_{\rm CM})]$. In particular, this is achieved when using

$$f_1(x_{\rm CM}) \quad = \quad 2(1-\gamma)\frac{\partial}{\partial x_{\rm CM}} D(x_{\rm CM}) \tag{4}$$

With the aforementioned choice of f_1, the Fokker-Planck equation is not a function of γ[9]

$$\frac{\partial}{\partial t} P(x_{\rm CM}, t) = \frac{\partial}{\partial x_{\rm CM}} D(x_{\rm CM}) \left(\beta \frac{\partial F(x_{\rm CM})}{\partial x_{\rm CM}} + \frac{\partial}{\partial x_{\rm CM}} \right) P(x_{\rm CM}, t)\,. \tag{5}$$

From Eq. 5, we then calculate the drifting velocity as follows

$$\begin{aligned} &\int {\rm d}x_{\rm CM} \cdot v_{\rm CM}(x_{\rm CM}) P(x_{\rm CM}, t) \equiv \int {\rm d}x_{\rm CM} \cdot x_{\rm CM} \frac{\partial}{\partial t} P(x_{\rm CM}, t) \\ = \quad &\int {\rm d}x_{\rm CM} \left[\frac{\partial}{\partial x_{\rm CM}} D(x_{\rm CM}) - \beta D(x_{\rm CM}) \frac{\partial F(x_{\rm CM})}{\partial x_{\rm CM}} \right] P(x_{\rm CM}, t)\,. \end{aligned} \tag{6}$$

In particular,

$$v_{\rm CM}(x_{\rm CM}) = v_x^{\rm FD}(x_{\rm CM}) + v_{x,\gamma}^{\rm FD}(x_{\rm CM})$$
$$v_{x,\gamma}^{\rm FD}(x_{\rm CM}) = \tfrac{\partial}{\partial x_{\rm CM}} D(x_{\rm CM}) \qquad v_x^{\rm FD}(x_{\rm CM}) = -\beta D(x_{\rm CM}) \tfrac{\partial F(x_{\rm CM})}{\partial x_{\rm CM}} \tag{7}$$

It is important to stress that Eq. 2 has not been derived using a rigorous bottom–up coarse–grained procedure. The latter is expected to result in a more general Langevin equation.[10,11] Moreover, the systematic coarse-graining of our current simulation model is complicated by the fact that our system is not Hamiltonian.

We now adapt the previous expression of the drifting velocity to our model. We recently estimated the diffusion constant $D(x_{\rm CM})$ of particles with mobile receptors[7] as

$$D(x_{\rm CM}) = c \frac{k_{\rm off}}{\langle n_{\rm b}(x_{\rm CM}) \rangle^2}, \tag{8}$$

where $x_{\rm CM}$ is the particle's center of mass, $n_{\rm b}$ the number of bridges, and $c = \pi R^2/2$ when away from the saturation condition ($n_{\rm b} < N_{\rm L}$ and $n_{\rm b} < N_{\rm R}$, where $N_{\rm L}$ is the number of ligands underneath the vesicle), with the vesicle radius equal to R. First, we calculate $v_x^{\rm FD}$ (Eq. 7) and then show that $v_{x,\gamma}^{\rm FD}$ is negligible compared to $v_x^{\rm FD}$. In our system, $F(x_{\rm CM})$ is the free energy of the system at a given $x_{\rm CM}$[8]

$$\beta F(x_{\rm CM}) = N_{\rm R} \log \left[1 - \frac{n_{\rm b}(x_{\rm CM})}{N_{\rm R}} \right] + N_{\rm L}(x_{\rm CM}) \log \left[1 - \frac{n_{\rm b}(x_{\rm CM})}{N_{\rm L}(x_{\rm CM})} \right] + n_{\rm b}(x_{\rm CM}) \,, \tag{9}$$

where $N_{\rm R}$ is the total number of receptors over the vesicle membrane. We have (see Sec. 2.4)

$$-\beta \frac{{\rm d}F(x_{\rm CM})}{{\rm d}x_{\rm CM}} = -N_{\rm L}'(x_{\rm CM}) \log \left[1 - \frac{n_{\rm b}(x_{\rm CM})}{N_{\rm L}(x_{\rm CM})} \right] \tag{10}$$

from which we derive

$$v_x^{\mathrm{FD}}(x_{\mathrm{CM}}) \;=\; -\frac{\pi R^2 k_{\mathrm{off}}}{2 n_{\mathrm{b}}(x_{\mathrm{CM}})^2} N_{\mathrm{L}}(x_{\mathrm{CM}})' \log\left[1 - \frac{n_{\mathrm{b}}(x_{\mathrm{CM}})}{N_{\mathrm{L}}(x_{\mathrm{CM}})}\right] \tag{11}$$

For a linear gradient of ligands ($\rho(x) = c_0 + \lambda x$, if ρ is the ligands' density) we have $N'_{\mathrm{L}}(x_{\mathrm{CM}}) = \lambda \pi R^2$. Therefore

$$\begin{aligned} v_x^{\mathrm{FD}}(x_{\mathrm{CM}}) &= -\frac{\lambda k_{\mathrm{off}}}{2}\left[\frac{\pi R^2}{n_{\mathrm{b}}(x_{\mathrm{CM}})}\right]^2 \log\left[1 - \frac{n_{\mathrm{b}}(x_{\mathrm{CM}})}{N_{\mathrm{L}}(x_{\mathrm{CM}})}\right] &(12)\\ &= -\frac{\lambda k_{\mathrm{off}}}{2}\left[\frac{\pi R^2}{n_{\mathrm{b}}(x_{\mathrm{CM}})}\right]^2 \log\left[1 - \frac{n_{\mathrm{b}}(x_{\mathrm{CM}})}{\pi R^2 \rho(x_{\mathrm{CM}})}\right] &(13)\\ &= -\frac{\lambda k_{\mathrm{off}}}{2\rho_{\mathrm{b}}(x_{\mathrm{CM}})^2} \log\left[1 - \frac{\rho_{\mathrm{b}}(x_{\mathrm{CM}})}{\rho(x_{\mathrm{CM}})}\right] &(14) \end{aligned}$$

where $\rho_{\mathrm{b}}(x_{\mathrm{CM}})$ is the density of bridges at the particle's center of mass and ρ is the density of ligands. The last equality in the previous equation follows from the fact that, for a given x_{CM}, $\rho_{\mathrm{b}}(x)$ is proportional to $\rho(x)$ (see Eq. 30). In the weak binding limit [$\rho_{\mathrm{b}}(x_{\mathrm{CM}}) \ll \rho(x_{\mathrm{CM}})$], v_x^{FD} further simplifies as follows

$$v_x^{\mathrm{FD}}(x_{\mathrm{CM}}) = \frac{\lambda k_{\mathrm{off}}}{2\rho_{\mathrm{b}}(x_{\mathrm{CM}})\rho(x_{\mathrm{CM}})} \,. \tag{15}$$

Note that the weak-binding regime is not relevant to the experimental conditions used in this work.

We now estimate $v_{x,\gamma}^{\mathrm{FD}}$ (Eq. 7)

$$v_{x,\gamma}^{\mathrm{FD}}(x_{\mathrm{CM}}) = D'(x_{\mathrm{CM}}) = -2\frac{\pi R^2 k_{\mathrm{off}}}{n_{\mathrm{b}}(x_{\mathrm{CM}})^3} n'_{\mathrm{b}}(x_{\mathrm{CM}}) \,. \tag{16}$$

Using Eq. 33 to express n'_b in terms of N'_L, we obtain

$$
\begin{aligned}
v^\mathrm{FD}_{x,\gamma}(x_\mathrm{CM}) &= -2\lambda k_\mathrm{off}\left[\frac{\pi R^2}{n_\mathrm{b}(x_\mathrm{CM})}\right]^2 \frac{N_\mathrm{R}-n_\mathrm{b}(x_\mathrm{CM})}{N_\mathrm{L}(x_\mathrm{CM})N_\mathrm{R}-n_\mathrm{b}(x_\mathrm{CM})^2} && (17)\\
&= -2\frac{\lambda k_\mathrm{off}}{\rho_\mathrm{b}(x_\mathrm{CM})^2}\frac{\rho_\mathrm{R}-\rho_\mathrm{b}(x_\mathrm{CM})}{\rho(x_\mathrm{CM})N_\mathrm{R}-\rho_\mathrm{b}(x_\mathrm{CM})n_\mathrm{b}(x_\mathrm{CM})} && (18)
\end{aligned}
$$

where ρ_R is the density of receptors calculated using the patch area (instead of the total area of the vesicle).

We now compare $v^\mathrm{FD}_{x,\gamma}(x_\mathrm{CM})$ with $v^\mathrm{FD}_x(x_\mathrm{CM})$. First, we consider the weak–binding regime (not relevant to this study) for which $\rho_\mathrm{b} \ll \rho \approx \rho_\mathrm{R}$. In this regime we find

$$
\frac{\rho_\mathrm{R}-\rho_\mathrm{b}(x_\mathrm{CM})}{\rho(x_\mathrm{CM})N_\mathrm{R}-\rho_\mathrm{b}(x_\mathrm{CM})n_\mathrm{b}(x_\mathrm{CM})} \approx \frac{1}{N_\mathrm{R}}\,, \tag{19}
$$

from which we derive

$$
\frac{|v^\mathrm{FD}_{x,\gamma}|}{|v^\mathrm{FD}_x|} \approx \frac{\rho}{\rho_\mathrm{b}N_\mathrm{R}}\,. \tag{20}
$$

The previous equality states that, at a given ρ_b/ρ, $v^\mathrm{FD}_{x,\gamma}$ readily becomes negligible when increasing the size of the vesicle (and therefore N_R at a given ρ_R). Interestingly, $v^\mathrm{FD}_{x,\gamma}$ may become important for small vesicles.

We then consider the regime (relevant to the present study) in which $\rho_\mathrm{b} \approx \rho < \rho_\mathrm{R}$ (see the linear regime in Fig. S17). In this case, we have

$$
\frac{\rho_\mathrm{R}-\rho_\mathrm{b}(x_\mathrm{CM})}{\rho(x_\mathrm{CM})N_\mathrm{R}-\rho_\mathrm{b}(x_\mathrm{CM})n_\mathrm{b}(x_\mathrm{CM})} \approx \frac{\rho_\mathrm{R}-\rho}{\rho(N_\mathrm{R}-n_\mathrm{b})}\,, \tag{21}
$$

and

$$
\frac{|v^\mathrm{FD}_{x,\gamma}|}{|v^\mathrm{FD}_x|} \approx \frac{\rho_\mathrm{R}-\rho}{\rho(N_\mathrm{R}-n_\mathrm{b})|\log(0^+)|}\,. \tag{22}
$$

where 0^+ is a small positive value. The previous equation implies that, for the vesicles considered in this work, $v_{x,\gamma}^{\mathrm{FD}}$ is negligible compared to v_x^{FD}. The drift velocity is thus given by v_x^{FD} (Eq. 14). It would be interesting to interpret this expression in the context of the balance between dissipation linked to the forces exerted by the bridges and the drag of the vesicle in a viscous medium, and the power generated by the gradient. However, such a calculation cannot be done in our current setting as we do not explicitly model the binding potential for bridges found at the end of the vesicle, but only consider geometric constraints due to the finite stretchability of the linkers.

Finally, it is instructive to consider the limit $\rho_{\mathrm{b}} \approx \rho_{\mathrm{R}} < \rho$ (see the constant regime in Fig. S17) in which the number of bridges is limited by the number of receptors present on the vesicle. In this case, Eq. 20 predicts $v_{x,\gamma}^{\mathrm{FD}}/k_{\mathrm{off}} = 0$ as the diffusion constant becomes uniform. On the contrary v_x^{FD} remains finite also in this limit.

2.3 Comparison between the drifting and the diffusive components of the motion

To check if the simulated and experimental drift/diffusion trajectories of the vesicles are in the regime described by Langevin dynamics, as postulated in Eq. 2, it is instructive to study the quantity $\delta = D/v_x^{\mathrm{FD}}$. This quantity has the dimensionality of a length, comparing the magnitudes of the diffusive and drifting components of the motion of the vesicles. From the results of the previous section, we calculate an expression of δ for Langevin trajectories as follows

$$\begin{aligned} \delta_{\mathrm{the}} &= \left(-\beta \frac{\partial F(x_{\mathrm{CM}})}{\partial x_{\mathrm{CM}}} + \frac{1}{D(x_{\mathrm{CM}})} \frac{\partial D(x_{\mathrm{CM}})}{\partial x_{\mathrm{CM}}} \right)^{-1} \approx \left(-\beta \frac{\partial F(x_{\mathrm{CM}})}{\partial x_{\mathrm{CM}}} \right)^{-1} \\ &= -\frac{1}{\lambda \pi R^2 \log\left(1 - \frac{\rho_{\mathrm{b}}}{\rho}\right)} \approx \frac{1}{\lambda R^2} , \end{aligned} \tag{23}$$

where the subscript "the" refers to "theoretical". Note that, for Langevin trajectories, δ_{the} is not a function of the diffusion constant (when neglecting effects of a non-uniform D) but depends only on the driving force given by the gradient of the free energy, F.

Using that $\lambda \approx 2 \cdot 10^{-7}\text{nm}^{-3}$, as in experiments, we estimate the order of magnitude of δ_{the} as follows

$$\frac{\delta_{\text{the}}}{\text{nm}} = \frac{5}{(R/\mu\text{m})^2} \tag{24}$$

This estimate predicts that, in experiments, δ should go from $\delta \approx 0.2\,\text{nm}$ to $\delta \approx 0.001\,\text{nm}$ when R increases from $R = 5\,\mu\text{m}$ to $R = 25\,\mu\text{m}$. An accurate comparison between experimental and theoretical estimates of δ is not possible given that experimental data for the diffusion component of the motion are too noisy and strongly influenced by localisation errors.

We can nonetheless provide a meaningful comparison between δ_{the} and its simulated counterpart δ_{sim}. In Fig. S26 we report the ratio $r = \delta_{\text{the}}/\delta_{\text{sim}}$ as a function of the density of bridges ρ_{b} for $R = 100\,\text{nm}$ (left) and $R = 500\,\text{nm}$ (right), using the simulation results of Figs. S19, S21. We find that $r \approx 0.3 - 0.9$ for $R = 100\,\text{nm}$ and deteriorates ($r \approx 0.01 - 0.09$) for bigger vesicles. r improves when decreasing ρ_{b}/ρ but it is not significantly impacted (within the current numerical precision) by the slope of the gradient λ.

Overall Fig. S26 suggests that the Langevin description of the vesicle motion Eq. 2 is semi-quantitative. The discrepancy between Langevin theory and simulations is controlled by vesicle size and bridge density, but we did not succeed in identifying a regime where agreement becomes fully quantitative within the numerically accessible range of parameters.

2.4 Proof of Eq. 10

2.4.1 Number and density of bridges

We first calculate the equilibrium predictions for the density and number of bridges ($\rho_{\rm b}(x_{\rm CM})$ and $n_{\rm b}(x_{\rm CM})$, respectively). The density of bridges underneath the particles at a given position x along the gradient solves the following equation

$$\rho_{\rm b}(x) = K_{\rm eq}\left[\rho(x)-\rho_{\rm b}(x)\right]\left[N_{\rm R}-n_{\rm b}(x_{\rm CM})\right] \qquad (25)$$

where $K_{\rm eq}$ is the equilibrium constant controlling the formation of bridges.[8] The number of bridges and the total number of ligands underneath the particles are obtained by integrating, respectively, $\rho_{\rm b}(x)$ and $\rho(x)$ over the contact region between the vesicle and the surface ($\Omega(x_{\rm CM})$)

$$n_{\rm b}(x_{\rm CM}) = \int_{\Omega(x_{\rm CM})} \rho_{\rm b}(x){\rm d}x{\rm d}y \qquad N_{\rm L}(x_{\rm CM}) = \int_{\Omega(x_{\rm CM})} \rho(x){\rm d}x{\rm d}y \qquad (26)$$

where y is the coordinate orthogonal to the gradient. By integrating Eq. 25 over $\Omega(x_{\rm CM})$ we find

$$n_{\rm b}(x_{\rm CM}) = K_{\rm eq}\left[N_{\rm L}(x_{\rm CM})-n_{\rm b}(x_{\rm CM})\right]\left[N_{\rm R}-n_{\rm b}(x_{\rm CM})\right] \qquad (27)$$

which is solved as follows

$$n_{\rm b}(x_{\rm CM}) = \frac{K_{\rm eq}N_{\rm L}(x_{\rm CM})+K_{\rm eq}N_{\rm R}+1-\sqrt{\Delta^2}}{2K_{\rm eq}} \qquad (28)$$

$$\Delta^2 = \left[K_{\rm eq}N_{\rm L}(x_{\rm CM})-K_{\rm eq}N_{\rm R}\right]^2+2K_{\rm eq}N_{\rm L}(x_{\rm CM})+2K_{\rm eq}N_{\rm R}+1 \qquad (29)$$

Given $n_{\rm b}(x_{\rm CM})$, the density of bridges reads as follows

$$\rho_{\rm b}(x) = \frac{K_{\rm eq}\left[N_{\rm R}-n_{\rm b}(x_{\rm CM})\right]}{1+K_{\rm eq}\left[N_{\rm R}-n_{\rm b}(x_{\rm CM})\right]}\rho(x) \qquad (30)$$

2.4.2 Drifting force

We now prove Eq. 10 using Eq. 27. From Eq. 9 we calculate

$$
\begin{aligned}
\beta F'(x_{\mathrm{CM}}) &= -\frac{n_{\mathrm{b}}'(x_{\mathrm{CM}})N_{\mathrm{R}}}{N_{\mathrm{R}}-n_{\mathrm{b}}(x_{\mathrm{CM}})}+\frac{n_{\mathrm{b}}(x_{\mathrm{CM}})N_{\mathrm{L}}'(x_{\mathrm{CM}})-N_{\mathrm{L}}(x_{\mathrm{CM}})n_{\mathrm{b}}'(x_{\mathrm{CM}})}{N_{\mathrm{L}}(x_{\mathrm{CM}})-n_{\mathrm{b}}(x_{\mathrm{CM}})}+n_{\mathrm{b}}'(x_{\mathrm{CM}}) \\
&\quad +N_{\mathrm{L}}'(x_{\mathrm{CM}})\log\left[1-\frac{n_{\mathrm{b}}(x_{\mathrm{CM}})}{N_{\mathrm{L}}(x_{\mathrm{CM}})}\right] \\
&\equiv \Lambda+N_{\mathrm{L}}'(x_{\mathrm{CM}})\log\left[1-\frac{n_{\mathrm{b}}(x_{\mathrm{CM}})}{N_{\mathrm{L}}(x_{\mathrm{CM}})}\right]
\end{aligned}
\tag{31}
$$

By deriving Eq. 27 we obtain

$$
\begin{aligned}
n_{\mathrm{b}}'(x_{\mathrm{CM}}) &= K_{\mathrm{eq}}\left[N_{\mathrm{L}}'(x_{\mathrm{CM}})-n_{\mathrm{b}}'(x_{\mathrm{CM}})\right]\left[N_{\mathrm{R}}-n_{\mathrm{b}}(x_{\mathrm{CM}})\right]-K_{\mathrm{eq}}\left[N_{\mathrm{L}}(x_{\mathrm{CM}})-n_{\mathrm{b}}(x_{\mathrm{CM}})\right]n_{\mathrm{b}}'(x_{\mathrm{CM}}) \\
&= \frac{n_{\mathrm{b}}(x_{\mathrm{CM}})\left[N_{\mathrm{L}}'(x_{\mathrm{CM}})-n_{\mathrm{b}}'(x_{\mathrm{CM}})\right]}{N_{\mathrm{L}}(x_{\mathrm{CM}})-n_{\mathrm{b}}(x_{\mathrm{CM}})}-\frac{n_{\mathrm{b}}(x_{\mathrm{CM}})n_{\mathrm{b}}'(x_{\mathrm{CM}})}{N_{\mathrm{R}}-n_{\mathrm{b}}(x_{\mathrm{CM}})}
\end{aligned}
\tag{32}
$$

where in the second line we have used Eq. 27. From the previous equation we obtain an expression for $n_{\mathrm{b}}'(x_{\mathrm{CM}})$ as a function of $N_{\mathrm{L}}'(x_{\mathrm{CM}})$

$$
n_{\mathrm{b}}'(x_{\mathrm{CM}}) = \frac{\left[N_{\mathrm{R}}-n_{\mathrm{b}}(x_{\mathrm{CM}})\right]n_{\mathrm{b}}(x_{\mathrm{CM}})N_{\mathrm{L}}'(x_{\mathrm{CM}})}{N_{\mathrm{L}}(x_{\mathrm{CM}})N_{\mathrm{R}}-n_{\mathrm{b}}(x_{\mathrm{CM}})^2}
\tag{33}
$$

By using the previous equation in Eq. 31 we can prove that the first three terms in the rhs of the latter cancel, therefore proving Eq. 10. Specifically

$$
\begin{aligned}
\Lambda &= -\frac{n_{\mathrm{b}}'(x_{\mathrm{CM}})N_{\mathrm{R}}}{N_{\mathrm{R}}-n_{\mathrm{b}}(x_{\mathrm{CM}})}+\frac{n_{\mathrm{b}}(x_{\mathrm{CM}})N_{\mathrm{L}}'(x_{\mathrm{CM}})-N_{\mathrm{L}}(x_{\mathrm{CM}})n_{\mathrm{b}}'(x_{\mathrm{CM}})}{N_{\mathrm{L}}(x_{\mathrm{CM}})-n_{\mathrm{b}}(x_{\mathrm{CM}})}+n_{\mathrm{b}}'(x_{\mathrm{CM}}) \\
&= -\frac{n_{\mathrm{b}}(x_{\mathrm{CM}})N_{\mathrm{R}}N_{\mathrm{L}}'(x_{\mathrm{CM}})}{N_{\mathrm{L}}(x_{\mathrm{CM}})N_{\mathrm{R}}-n_{\mathrm{b}}(x_{\mathrm{CM}})^2}+\frac{n_{\mathrm{b}}(x_{\mathrm{CM}})N_{\mathrm{L}}'(x_{\mathrm{CM}})}{N_{\mathrm{L}}(x_{\mathrm{CM}})-n_{\mathrm{b}}(x_{\mathrm{CM}})} \\
&\quad -\frac{N_{\mathrm{L}}(x_{\mathrm{CM}})}{N_{\mathrm{L}}(x_{\mathrm{CM}})-n_{\mathrm{b}}(x_{\mathrm{CM}})}\frac{\left[N_{\mathrm{R}}-n_{\mathrm{b}}(x_{\mathrm{CM}})\right]n_{\mathrm{b}}(x_{\mathrm{CM}})N_{\mathrm{L}}'(x_{\mathrm{CM}})}{N_{\mathrm{L}}(x_{\mathrm{CM}})N_{\mathrm{R}}-n_{\mathrm{b}}(x_{\mathrm{CM}})^2} \\
&\quad +\frac{\left[N_{\mathrm{R}}-n_{\mathrm{b}}(x_{\mathrm{CM}})\right]n_{\mathrm{b}}(x_{\mathrm{CM}})N_{\mathrm{L}}'(x_{\mathrm{CM}})}{N_{\mathrm{L}}(x_{\mathrm{CM}})N_{\mathrm{R}}-n_{\mathrm{b}}(x_{\mathrm{CM}})^2} \\
&= \frac{n_{\mathrm{b}}(x_{\mathrm{CM}})N_{\mathrm{L}}'(x_{\mathrm{CM}})}{N_{\mathrm{L}}(x_{\mathrm{CM}})-n_{\mathrm{b}}(x_{\mathrm{CM}})}-\frac{N_{\mathrm{L}}(x_{\mathrm{CM}})}{N_{\mathrm{L}}(x_{\mathrm{CM}})-n_{\mathrm{b}}(x_{\mathrm{CM}})}\frac{\left[N_{\mathrm{R}}-n_{\mathrm{b}}(x_{\mathrm{CM}})\right]n_{\mathrm{b}}(x_{\mathrm{CM}})N_{\mathrm{L}}'(x_{\mathrm{CM}})}{N_{\mathrm{L}}(x_{\mathrm{CM}})N_{\mathrm{R}}-n_{\mathrm{b}}(x_{\mathrm{CM}})^2} \\
&\quad -\frac{n_{\mathrm{b}}(x_{\mathrm{CM}})^2N_{\mathrm{L}}'(x_{\mathrm{CM}})}{N_{\mathrm{L}}(x_{\mathrm{CM}})N_{\mathrm{R}}-n_{\mathrm{b}}(x_{\mathrm{CM}})^2} \\
&= \frac{n_{\mathrm{b}}(x_{\mathrm{CM}})N_{\mathrm{L}}'(x_{\mathrm{CM}})}{N_{\mathrm{L}}(x_{\mathrm{CM}})-n_{\mathrm{b}}(x_{\mathrm{CM}})} \\
&\quad -\frac{n_{\mathrm{b}}(x_{\mathrm{CM}})N_{\mathrm{L}}'(x_{\mathrm{CM}})}{N_{\mathrm{L}}(x_{\mathrm{CM}})N_{\mathrm{R}}-n_{\mathrm{b}}(x_{\mathrm{CM}})^2}\left[\frac{N_{\mathrm{L}}(x_{\mathrm{CM}})\left[N_{\mathrm{R}}-n_{\mathrm{b}}(x_{\mathrm{CM}})\right]}{N_{\mathrm{L}}(x_{\mathrm{CM}})-n_{\mathrm{b}}(x_{\mathrm{CM}})}+n_{\mathrm{b}}(x_{\mathrm{CM}})\right] \\
&= \frac{n_{\mathrm{b}}(x_{\mathrm{CM}})N_{\mathrm{L}}'(x_{\mathrm{CM}})}{N_{\mathrm{L}}(x_{\mathrm{CM}})-n_{\mathrm{b}}(x_{\mathrm{CM}})}-\frac{n_{\mathrm{b}}(x_{\mathrm{CM}})N_{\mathrm{L}}'(x_{\mathrm{CM}})}{N_{\mathrm{L}}(x_{\mathrm{CM}})N_{\mathrm{R}}-n_{\mathrm{b}}(x_{\mathrm{CM}})^2}\frac{N_{\mathrm{L}}(x_{\mathrm{CM}})N_{\mathrm{R}}-n_{\mathrm{b}}(x_{\mathrm{CM}})^2}{N_{\mathrm{L}}(x_{\mathrm{CM}})-n_{\mathrm{b}}(x_{\mathrm{CM}})} \\
&= 0
\end{aligned}
$$

3 Supplementary results from statistical testing

3.1 Statistical significance of vesicle drift

To investigate the statistical significance of vesicle drift along the gradient direction, we have employed the one-sample (two-sided) Student's t-test, testing the null hypothesis that the mean of a distribution is equal to zero. Note that the t-test is robust to non-normality of data when the sample sizes are sufficiently large;[12] the t-test requires that the sample means are normally distributed, and the central limit theorem states that the distribution of sample means approximates a normal distribution as the sample size gets larger. Our simulated samples consist of 1000 vesicle trajectories for each combination of sticky end length and vesicle size; meanwhile, our experimental sample sizes vary with sticky end length (due to random fluctuations in vesicle counts between different experimental runs) and vesicle size (due to non-uniform size distributions), but are generally on the order of hundreds of vesicles.

If we apply this test to the experimental data for $l = 5$ shown in the scatter plots in Fig. 2a, the t-test on the data for vesicle velocities *parallel* to the gradient gives a p-value of 6.96×10^{-25}, while the t-test on the data for vesicle velocities *perpendicular* to the gradient gives a p-value of 0.377. Regarding experimental data for $l = 6$ shown in the scatter plots in Fig. 2a, applying the t-test to vesicle velocities parallel to the gradient gives a p-value of 2.44×10^{-4}, while the t-test on vesicle velocities perpendicular to the gradient gives a p-value of 0.783. Finally, regarding the experimental data for $l = 7$ shown in the scatter plots in Fig. 2a, the t-test yields a p-value of 0.607 when applied to vesicle velocities parallel to the gradient, and a p-value of 0.546 for vesicle velocities perpendicular to the gradient. The results from the t-tests demonstrate statistical significance for motion parallel to the gradient in the $l = 5$ and $l = 6$ system, *i.e.* the mean vesicle velocity parallel to the gradient is non-zero. This is not the case for the $l = 7$ system, however. As discussed in the main text, due to the increased binding strength of the longer sticky ends, the mobility of vesicles is reduced such that their motion (within the timescale of experiments) is indistinguishable

from tracking errors. Regarding motion perpendicular to the gradient, the t-test results indicate that the mean vesicle velocity perpendicular to the gradient is likely to be zero, for each of the $l = 5$, 6 and 7 nt systems.

We now turn our attention to the equivalent data from simulations (plotted in Fig. 2b); here we discuss the results for the simulations of vesicles with 1 μm diameter. For $l = 5$, the t-test yields p-values of 4.69×10^{-4} and 0.683 for vesicle velocities parallel and perpendicular to the gradient, respectively. For $l = 6$, the t-test yields p-values of 3.89×10^{-6} and 0.545, for vesicle velocities parallel and perpendicular to the gradient. Finally, for $l = 7$, the t-test yields p-values of 0.0101 and 0.824, for vesicle velocities parallel and perpendicular to the gradient. In each of the $l = 5$, 6 and 7 systems, the t-test results show that the mean vesicle velocity parallel to the gradient is significantly different to zero, whereas the data for vesicle velocities perpendicular to the gradient is consistent with having zero mean.

To further test that the vesicle drift parallel to the gradient is in the direction of increasing ligand density, we now conduct the one-sample *one-sided* Student's t-test. This tests the null hypothesis that the mean velocity parallel to the gradient is less than or equal to zero. Referring to the same set of data as above, for experimental vesicles, the t-test yields p-values of 3.48×10^{-25}, 1.22×10^{-4} and 0.697 for $l = 5$, 6 and 7, respectively. For simulation results, the t-test yields p-values of 2.35×10^{-4}, 1.95×10^{-6} and 5.06×10^{-3} for $l = 5$, 6 and 7, respectively. The results indicate statistical significance in the observation that vesicles drift along the gradient in the direction of increasing ligand density on average, except for the case of $l = 7$ in experiments (which is likely due to the low signal-to-noise ratio).

Altogether, the findings from the statistical significance tests on experimental and simulated vesicle motion support the conclusions outlined in the main text: vesicles preferentially drift in the direction parallel to the local ligand density gradient.

3.2 Relationship between vesicle diameter and drift velocity

To further investigate the relationship between vesicle diameter and drift velocity, we have conducted statistical significance tests.

We proceed with the one-sided Welch's t-test, which tests the null hypothesis that the mean of a population if significantly greater than the mean of another population; unlike the Student's t-test, it does not assume that the populations have equal variance. We use this to compare the mean displacements of vesicles binned by vesicle diameter; in this case, the null hypothesis is that the mean displacement of the bin corresponding to larger vesicles is less than or equal to the mean displacement of the other bin. Referring to the experimental data for the $l = 5$ system plotted in Fig. 3 in the main text (average vesicle velocity, binned by vesicle diameter), if we conduct Welch's t-test on the smallest ($< 5\,\mu$m) and largest ($> 25\,\mu$m) diameter bins, we obtain a p-value of 7.37×10^{-6}. Alternatively, if we split the vesicle data from the $l = 5$ experiments into two groups, one with vesicle diameter $< 15\,\mu$m, and the other with vesicle diameter $> 15\,\mu$m, Welch's t-test on these two groups gives a p-value of 1.19×10^{-6}. Turning our attention to the other sticky end length systems, if we split the vesicle data from the $l = 6$ experiments into two groups (the first with vesicle diameters $< 15\,\mu$m, the second with diameters $> 15\,\mu$m), Welch's t-test returns a p-value of 2.30×10^{-3}. For $l = 7$, this same test returns a p-value of 0.981.

We also conduct the same statistical testing on the simulation data plotted in Fig. 3, comparing the average velocity of the vesicles with $0.2\,\mu$m diameter versus vesicles with $1.8\,\mu$m diameter. The one-sided Welch's t-test yields p-values of 1.17×10^{-2}, 9.32×10^{-3} and 0.359 for $l = 5$, 6 and 7, respectively.

For both experimental and simulated trajectories, the results of the t-test indicate that we can reject the null hypothesis for $l = 5$ and $l = 6$ with statistical significance. This would mean that larger vesicles drift at a significantly greater velocity parallel to the gradient than smaller vesicles, and that they drift in the direction of increasing ligand density. Note that this does not hold for $l = 7$, although this is likely due to the drift velocity being so low that

it is indistinguishable from the stochastic component of the motion and/or tracking errors for experimental trajectories, as mentioned in the main text. These statistical observations support our conclusion that vesicle size impacts drift velocity along the ligand concentration gradient.

4 Supplementary tables

Table S1: Sequences of all DNA strands used in this work (5' to 3'). The substrate-anchored ligand constructs were manufactured from equal parts of strands S1, S5 and S6, while the membrane-anchored receptor constructs were manufactured from equal parts of strands S2, S3 and S4, with S3. Strands S2 and S5 feature complementary sticky ends, and sequences are displayed for systems with sticky end lengths varying from three to ten bases. The ligand and receptor constructs feature biotin and cholesterol/cholesteryl modifications respectively for anchoring to the relevant surfaces. For fluorescent labelling of the ligands and receptors, strands S1 and S2 are labelled with the fluorophore Alexa Fluor 488. Schematics of the constructs are depicted in Figure S2.

**Construct**	**Strand**	**Sticky end length / nt**	**Sequence (5' to 3')**
Ligand	S1	3–10	CGCGACTTCCTCGCCGCG CGCGAGTTCGAGCTACGC (—AF488)
	S5	3	GCG TT GCGTAGCTCGAACTCGCG
		4	GCGG TT GCGTAGCTCGAACTCGCG
		5	GCGGC TT GCGTAGCTCGAACTCGCG
		6	GCGTGC TT GCGTAGCTCGAACTCGCG
		7	CGCACCG TT GCGTAGCTCGAACTCGCG
		8	GTGGACGC TT GCGTAGCTCGAACTCGCG
		9	GGTCGCAGC TT GCGTAGCTCGAACTCGCG
		10	CGTCCGTGCC TT GCGTAGCTCGAACTCGCG
	S6	3–10	CGCGGCGAGGAAGTCGCG — Biotin
Receptor	S2	3	CGC TT GAGAGTAGGACCGGCGCG
		4	CCGC TT GAGAGTAGGACCGGCGCG
		5	GCCGC TT GAGAGTAGGACCGGCGCG
		6	GCACGC TT GAGAGTAGGACCGGCGCG
		7	CGGTGCG TT GAGAGTAGGACCGGCGCG (—AF488)
		8	GCGTCCAC TT GAGAGTAGGACCGGCGCG
		9	GCTGCGACC TT GAGAGTAGGACCGGCGCG
		10	GGCACGGACG TT GAGAGTAGGACCGGCGCG
	S3	3–10	CGTTTGCAGGAACGAGAC TT — Cholesterol-TEG
	S4	3–10	Cholesteryl-TEG — TT GTCTCGTTCCTGCAAACG CGCGCCGGTCCTACTCTC

5 Supplementary figures

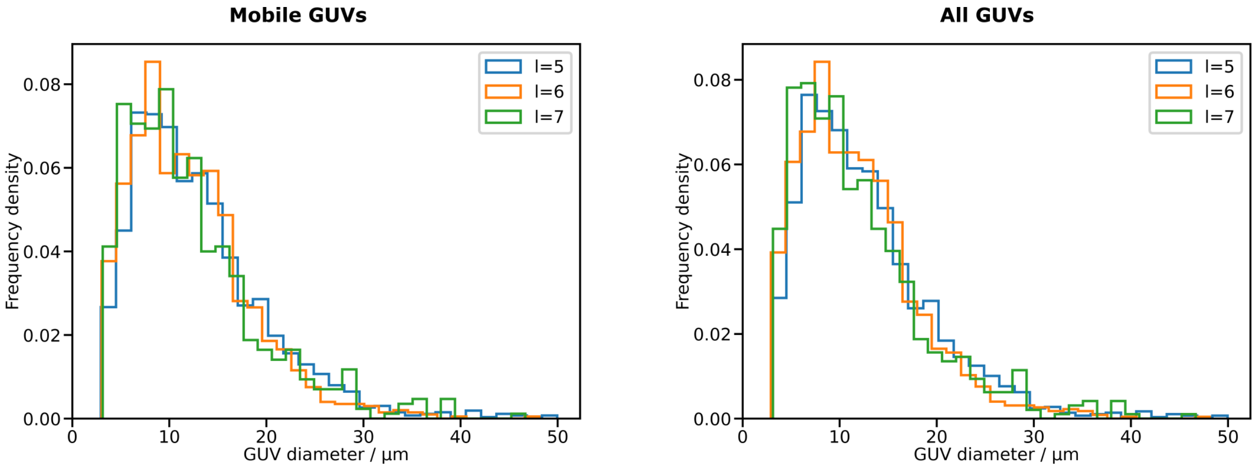

Figure S1: **Size distributions of vesicles recorded in experiments.** Frequency-density histograms are shown for the filtered (mobile) vesicle population (left) and for all vesicles (right). The filtering method is outlined in Section 1.4. The vesicles have been produced *via* electroformation, which generates polydisperse samples with median diameters ranging between 10–11 μm and mean diameters ranging between 12–13 μm. Note that as part of our vesicle tracking algorithm, we exclude vesicles with diameters measured to be less than 2 μm, to avoid erroneous tracking of noise or bright specks in the image. The method of tracking the vesicles and measuring their diameters is further detailed in Section 1 of the SI.

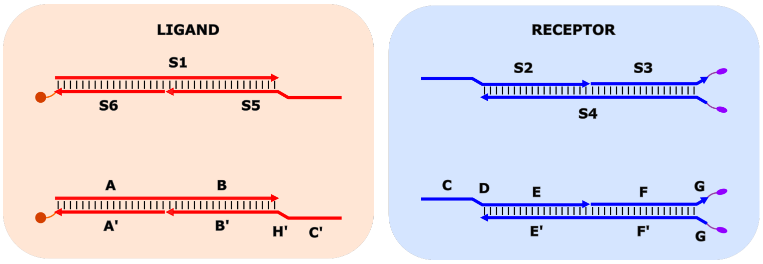

Figure S2: **Schematics of the DNA ligand and receptor constructs used in this work, labelled by their constituent strands (top) and by their domain structure (bottom).** Domains are separated by white spaces in the corresponding sequences of Table S1. Domains C and C' are complementary sticky ends.

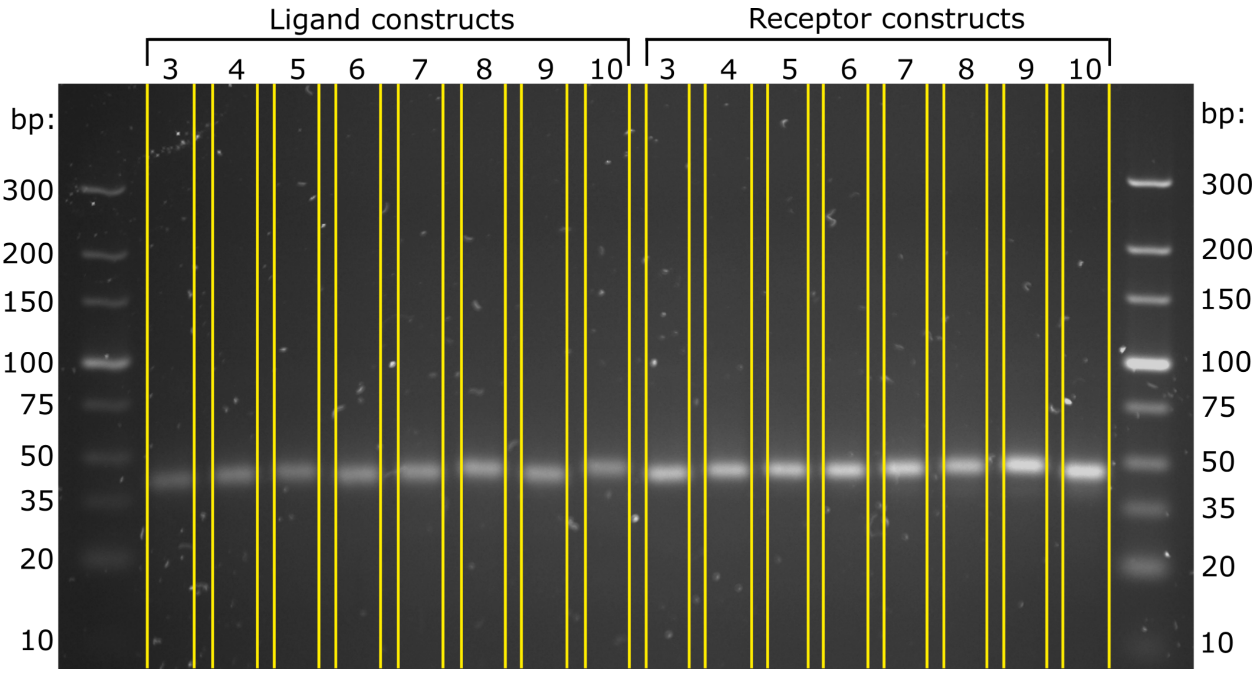

Figure S3: **Gel electrophoresis results for DNA ligand and receptor constructs.** Results for ligands and receptors with sticky end lengths $l = 3$–10 nt are shown. A sharp peak between the 35 and 50 bp reference markers of the DNA ladders is observed for all constructs, which agrees with the expected construct sizes.

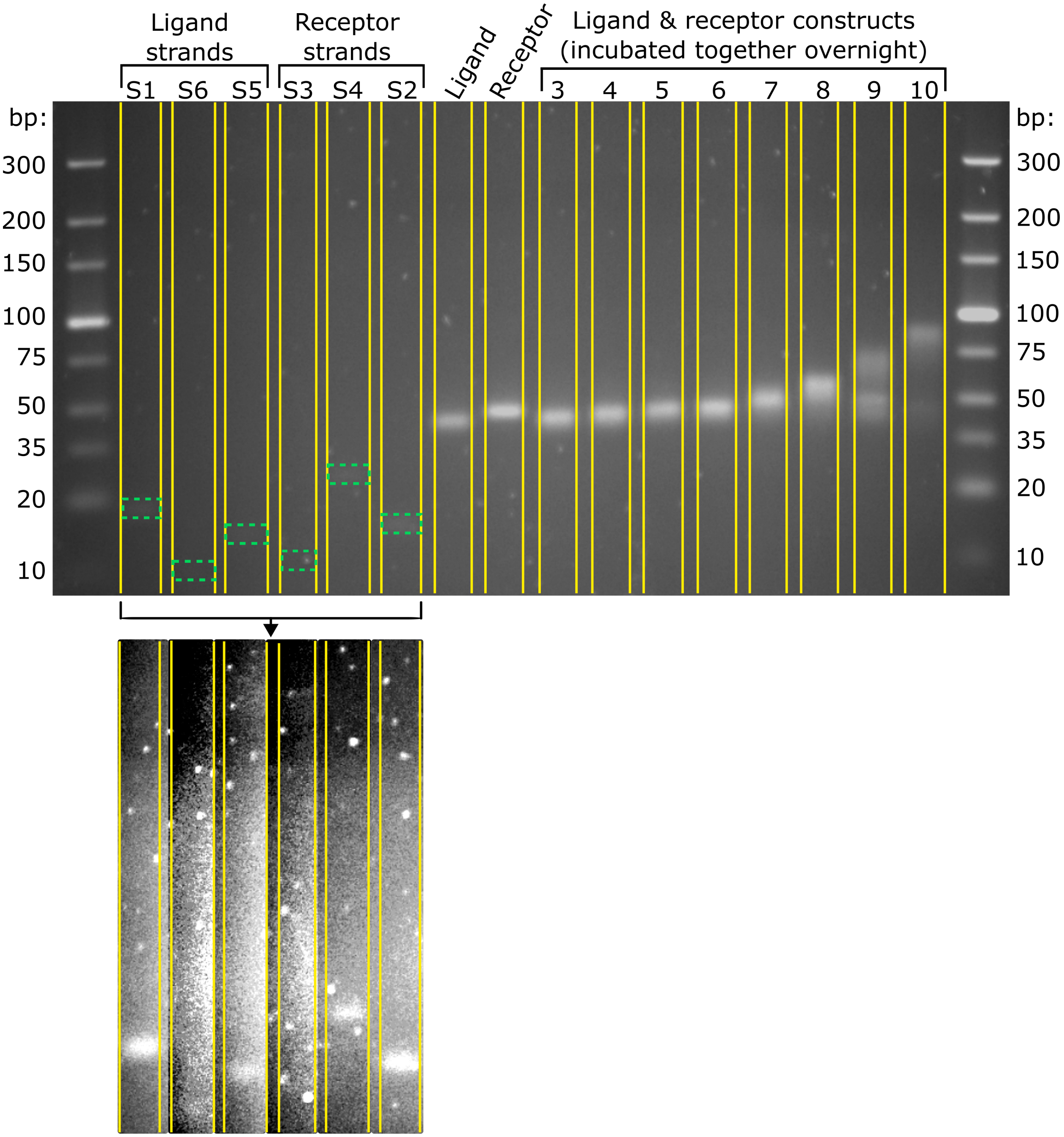

Figure S4: **Gel electrophoresis results verifying formation of ligand and receptor constructs, and increased binding strength between complementary sticky ends as sticky end length increases.** On the left half of the gel, results are shown for individual ligand and receptor strands (S1–6) and annealed constructs with sticky end lengths $l = 5\,\text{nt}$. As the gel stain (SYBR Safe) preferentially binds to double-stranded DNA, the bands for the individual strands are significantly fainter than the bands for the annealed constructs. The approximate positions of the single-stranded DNA bands have been annotated with dashed boxes to aid visualisation, and this section of the gel is shown with enhanced lightness-contrast settings (bottom). On the right half of the gel, results are shown for samples where $1\,\mu\text{M}$ ligand and $1\,\mu\text{M}$ receptor were incubated together overnight, for $l = 3$–$10\,\text{nt}$; as l increases, the presence of a second, larger species (a ligand bound to a receptor) in the sample becomes more pronounced.

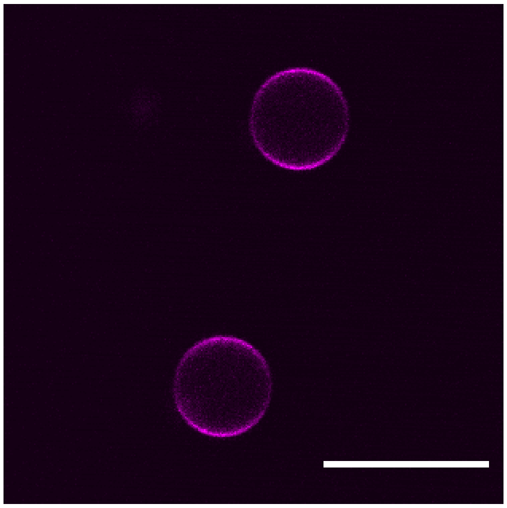

Figure S5: **Confocal image of two vesicles functionalised with DNA "receptor" constructs**. In this case, the fluorescence signal is from the Alexa Fluor 48 tag on the receptor constructs bound to the membrane. The receptors have a sticky end length of 7 nt. The scale bar indicates a length of 20 μm.

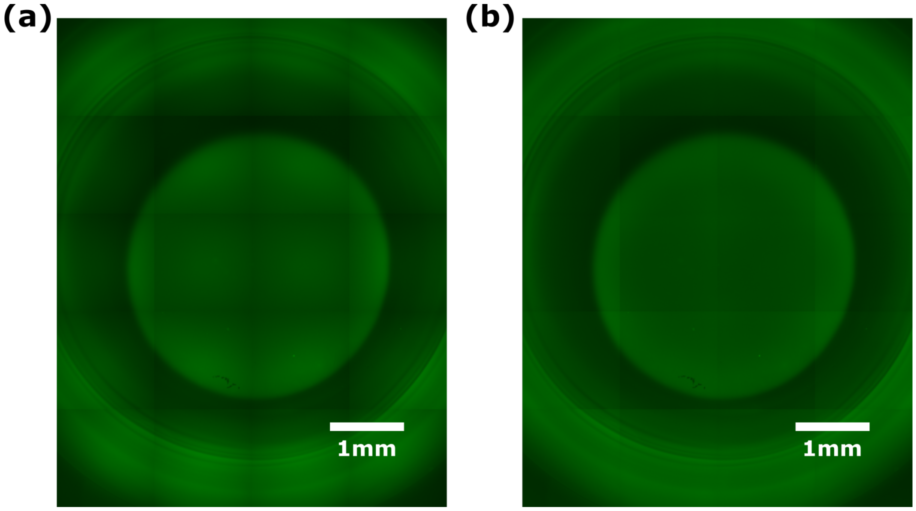

Figure S6: **Fluorescence images of ligand coverage of a well, before and after intensity normalisation.** The ligands constructs have been functionalised with the fluorophore Alexa Fluor 488, to enable visualisation of the DNA coverage. As described in the experimental methods section, a small circular region of the well has been functionalised with ligands, resulting in a steep ligand-density gradient at the perimeter of this region. The images are composed of multiple fields of view stitched together. Due to non-uniform illumination of the samples, the edges of the field of view have reduced fluorescence intensity, as can be seen in (a) with the images prior to normalisation. To account for this non-uniform illumination, the image intensities were normalised by scaling each field of view by a control image of a calcein solution; (b) shows the images post-normalisation.

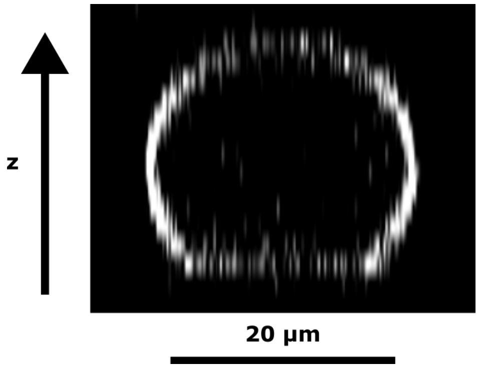

Figure S7: **Cross-sectional image of a vesicle adhered to a substrate.** The vesicle membrane has been functionalised with receptors, which bind to the complementary ligands anchored to a substrate. The lipid membrane of the vesicle also features a small proportion of fluorescently-labelled lipid molecules, allowing for the vesicle to be imaged with confocal microscopy. This cross-sectional view has been constructed from a confocal z-stack of the vesicle, and shows how the vesicle shape has been deformed to a truncated sphere with a flat contact region adhered to the substrate. Note that the diameter of the contact region is comparable (but slightly smaller than) the diameter of the vesicle; in our simulations and theoretical derivations we approximate the vesicle shape as a hemisphere.

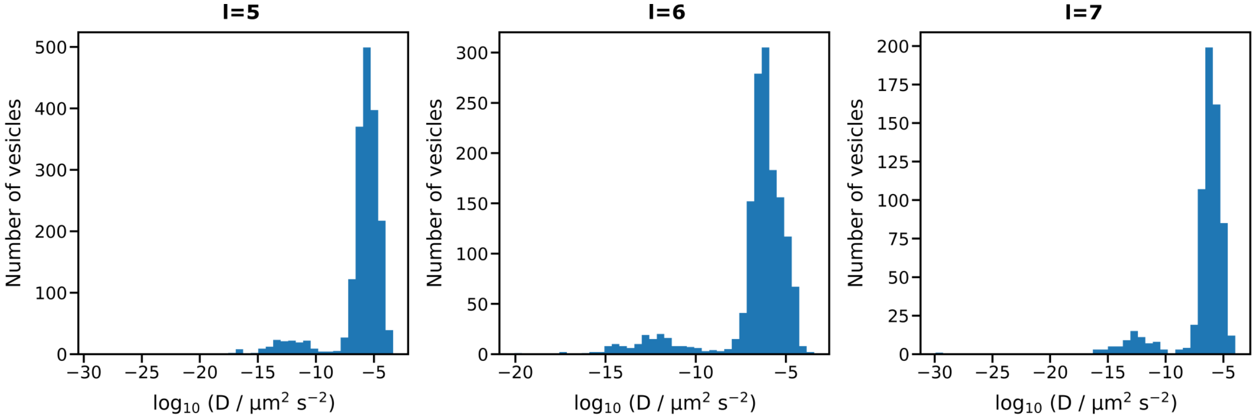

Figure S8: **Estimated diffusion coefficients of vesicles measured in experiments.** The histograms illustrate the distribution of diffusion coefficients, D, estimated for experimental systems with sticky end length $l = 5$, 6 and 7 nt. The diffusion coefficients were estimated from experimental trajectory data *via* the method outlined in Section 1.4 of the SI. By plotting the decimal logarithm of the vesicle diffusion coefficients on the x-axis, bimodal distributions of the estimated diffusion coefficients are visible.

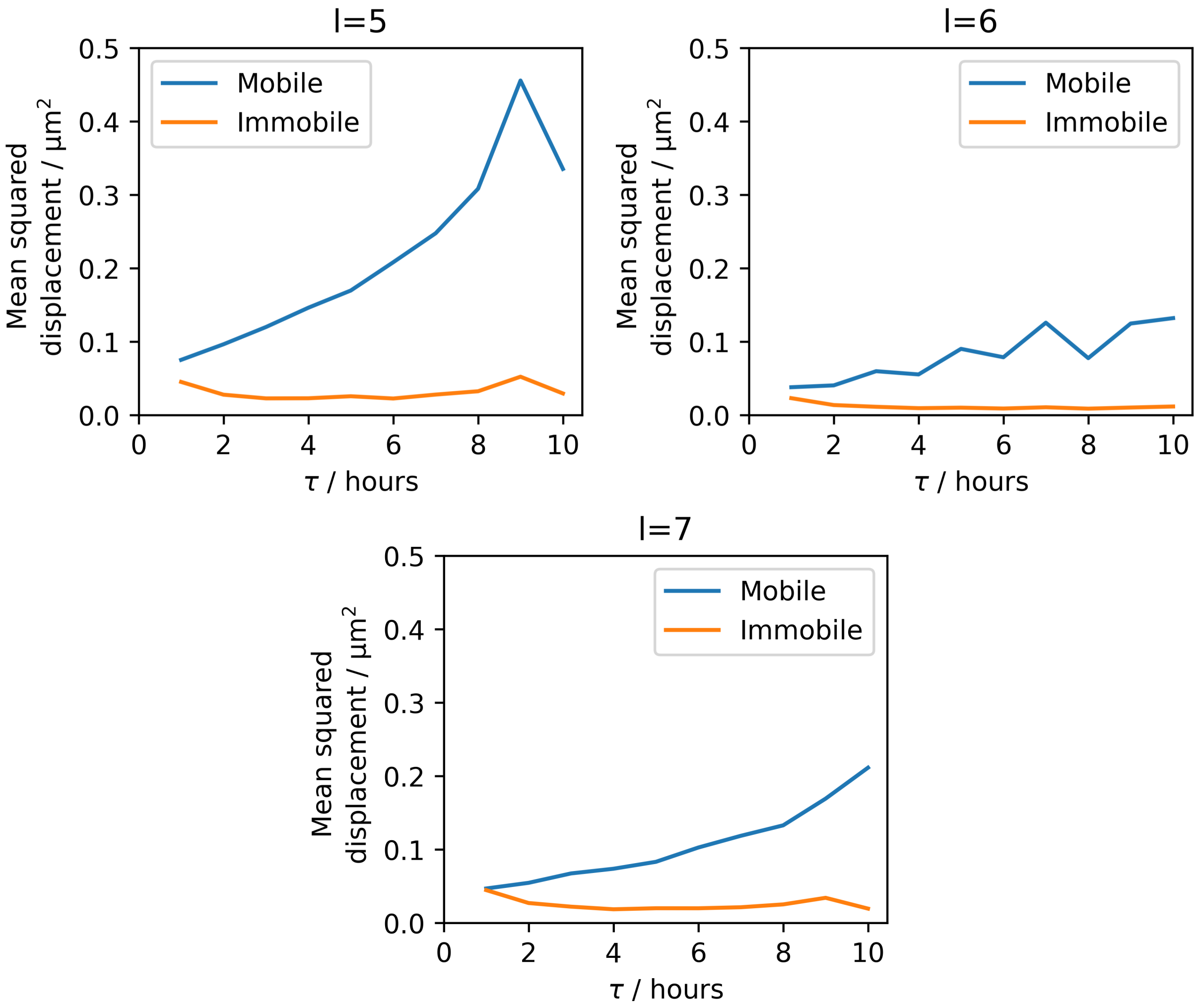

Figure S9: **Mean squared displacement (MSD) of mobile and immobile GUV populations.** Data are shown for the $l = 5$–$7\,$nt sticky end systems. The MSD of the immobile GUV populations show minimal motion in each of the three systems, while the MSDs of the mobile population increase with time. Note that MSD estimates become increasingly noisy with time due to worsening statistics.

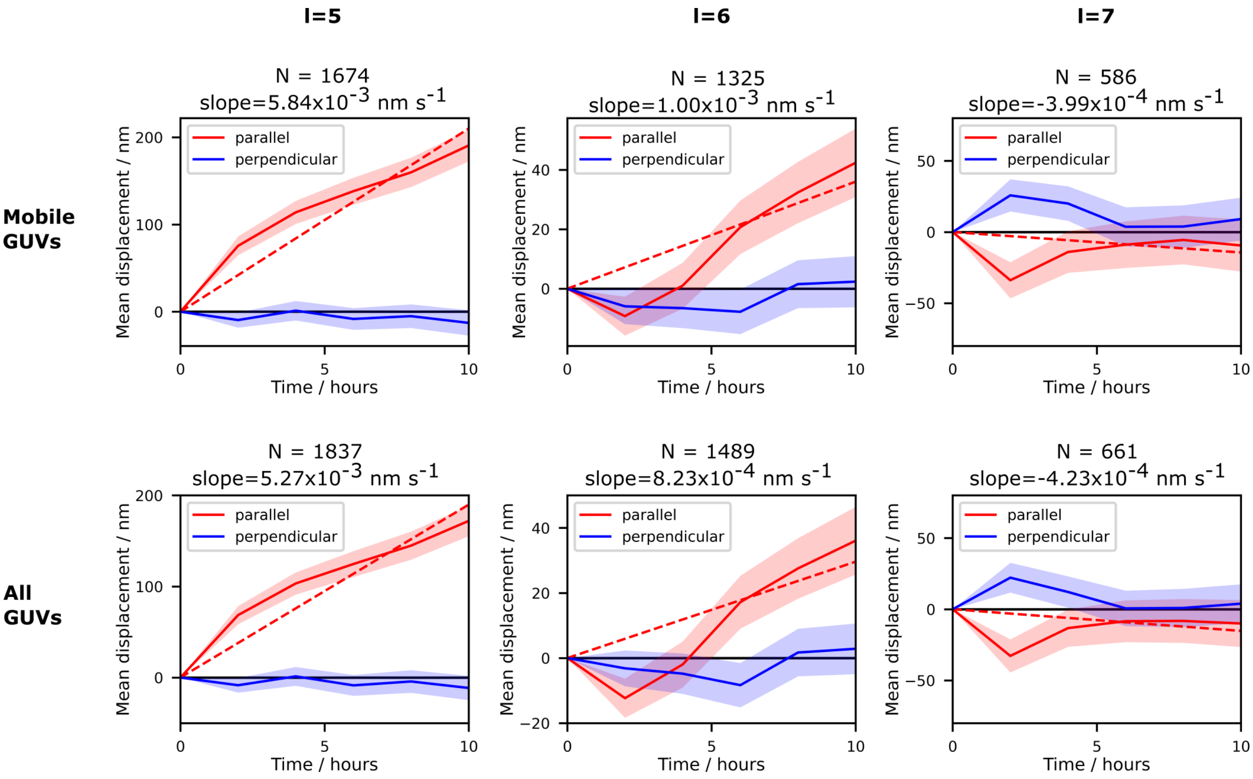

Figure S10: **Comparing experimental vesicle trajectories over time, for sticky end lengths l = 5, 6 and 7 nt.** The line plots illustrate the mean displacements of vesicles parallel and perpendicular to the gradient direction, where positive values of displacement parallel to the gradient indicate vesicle motion towards higher ligand density regions. Straight lines have been fitted to the data for motion parallel to the gradient, to extract the average vesicle drifting velocities. Each plot is annotated with the number N of vesicles averaged over, and the slope of the linear fit. Data are displayed for the filtered (mobile) vesicle population (top, also shown in Fig. 2) and for all vesicles (bottom); the filtering method is outlined in Section 1.4 of the SI.

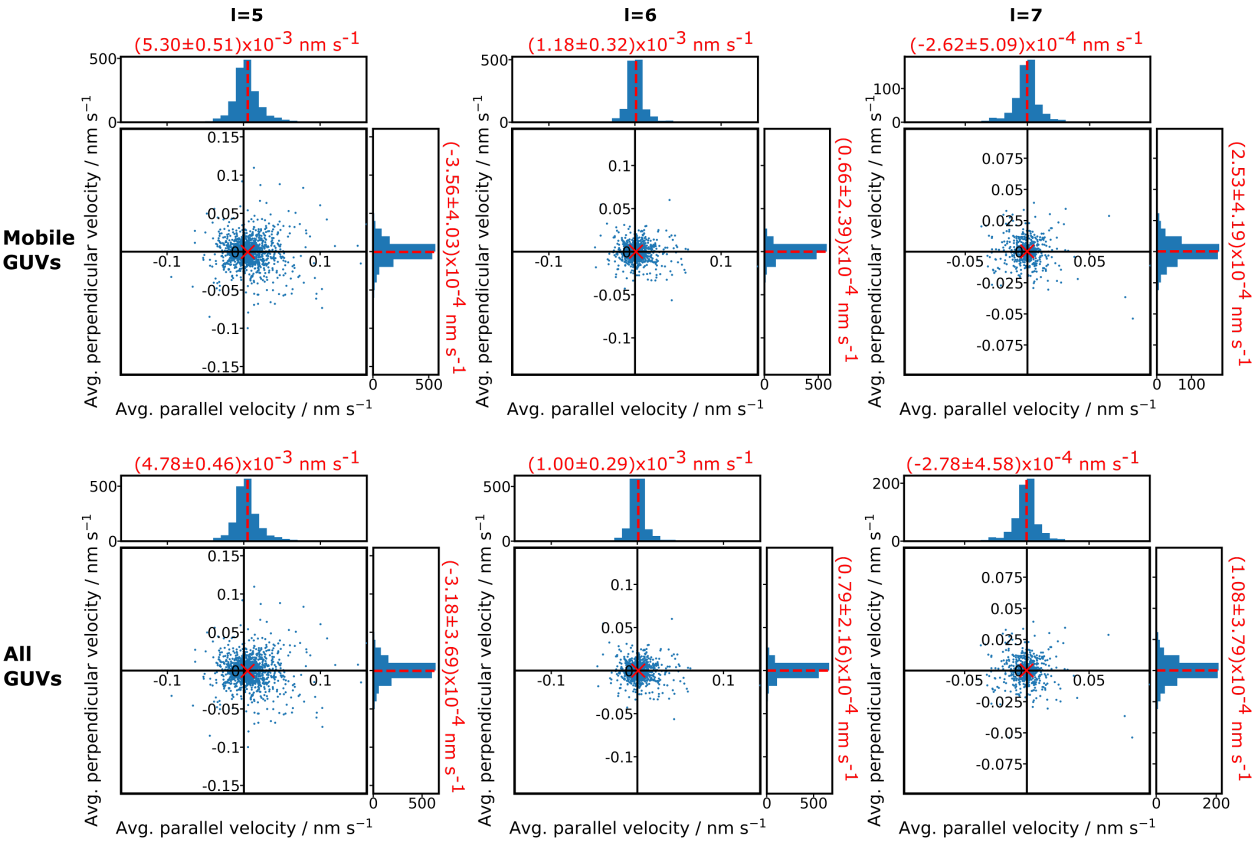

Figure S11: **Full distributions of velocities relative to the gradient direction, for vesicles measured in experiments.** The plots show experimental data for the distribution of vesicle velocities parallel and perpendicular to the gradient direction, where the velocity has been calculated as total displacement over the trajectory divided by the total duration. The data are for $l = 5$, 6 and 7 nt sticky end systems, and includes all outliers. Positive values of displacement parallel to the gradient indicate vesicle motion in the direction of increasing ligand density. Data are displayed for the filtered (mobile) vesicle population (top, also shown in Fig. 2) and all vesicles (bottom); the filtering method is outlined in Section 1.4. Note that the GUV counts per velocity bin differ between the histograms shown here and those in Fig. 2 of the main text. This is a consequence of using an equal number of bins in the two figures over a different range of velocities.

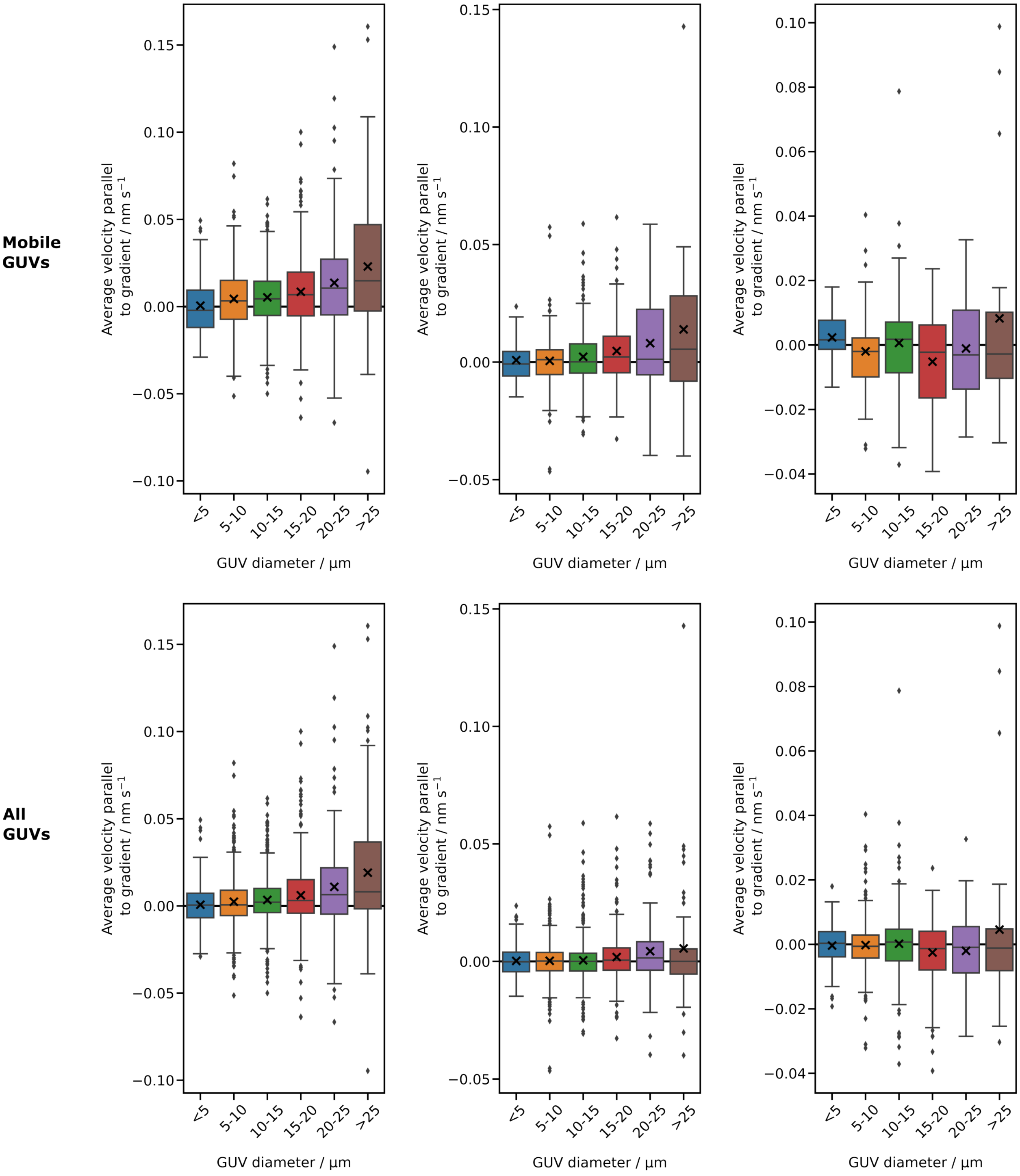

Figure S12: **Distribution of average vesicle velocity parallel to the gradient binned by vesicle size, for vesicles measured in experiments.** Data are shown for $l = 5$, 6 and 7 nt sticky end systems, and the distributions are shown for the filtered (mobile) vesicle population (top, also shown in Fig. 3) and for all vesicles (bottom); the filtering method is detailed in Section 1.4 of the SI. The average vesicle velocities have been calculated as total displacement divided by the trajectory duration, where positive velocity values indicate motion along the gradient in the direction of increasing ligand density. The mean of the distributions for each vesicle size range is marked with a cross, and all outliers have been included.

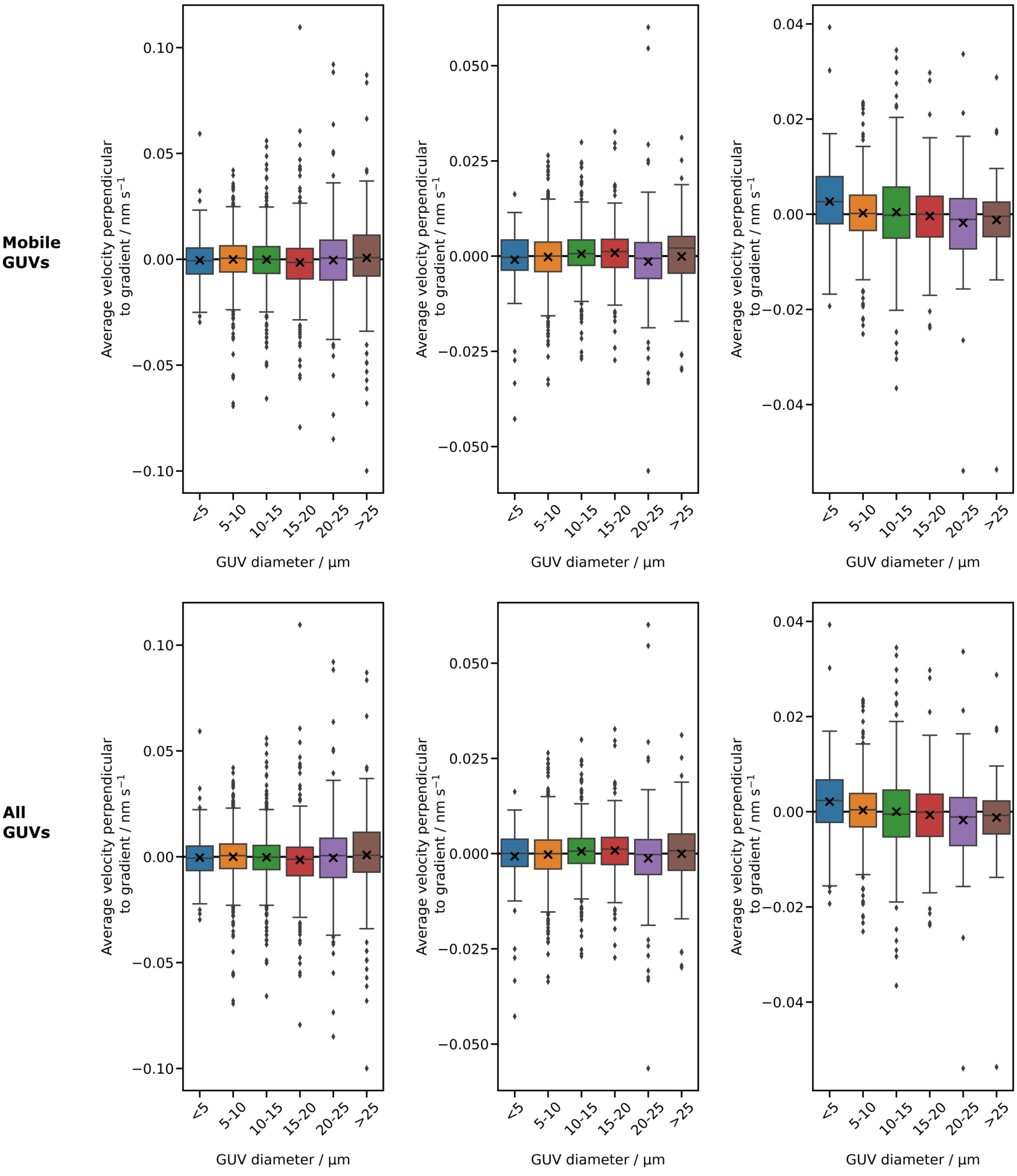

Figure S13: **Distribution of average vesicle velocity perpendicular to the gradient binned by vesicle size, for vesicles measured in experiments.** Data are shown for $l = 5$, 6 and 7 nt sticky end systems, and the distributions are shown for the filtered (mobile) vesicle population (top) and for all vesicles (bottom); the filtering method is detailed in Section 1.4 of the SI. The average vesicle velocities have been calculated as total displacement perpendicular to the gradient divided by the trajectory duration.The mean of the distributions for each vesicle size range is marked with a cross.

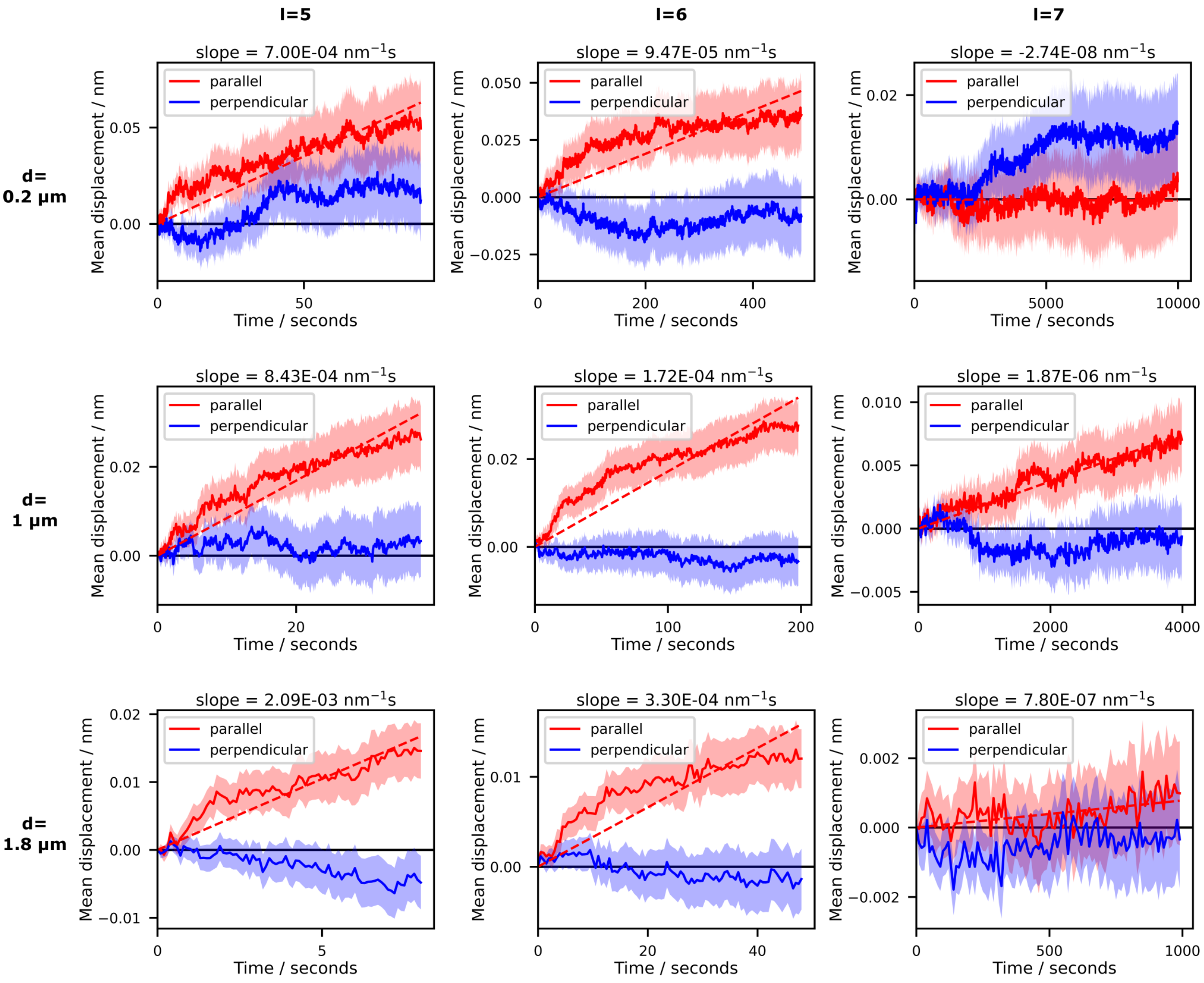

Figure S14: **Full trajectory data from simulations, for varying vesicle diameter and sticky end length.** The plots illustrate simulation results for mean vesicle displacement versus time, where the displacement has been decomposed into its components parallel and perpendicular to the gradient direction. The standard error of the mean displacement has been shaded. The data is for $l = 5$, 6, and 7 nt sticky end systems, with vesicle diameters $d = 0.2$, 1 and 1.8 μm. Positive values of displacement parallel to the gradient indicate vesicle motion in the direction of increasing DNA surface density on the substrate. Straight lines have been fitted to the data for motion parallel to the gradient, and each plot has been annotated with the slope of the linear fit. Data for $d = 1\,\mu$m are also shown in Fig. 2. Note that the apparent plateauing of mean displacement over time is likely due to vesicles hitting regions of the substrate with higher local ligand density, present due to the random process through which ligands are distributed on the surface.

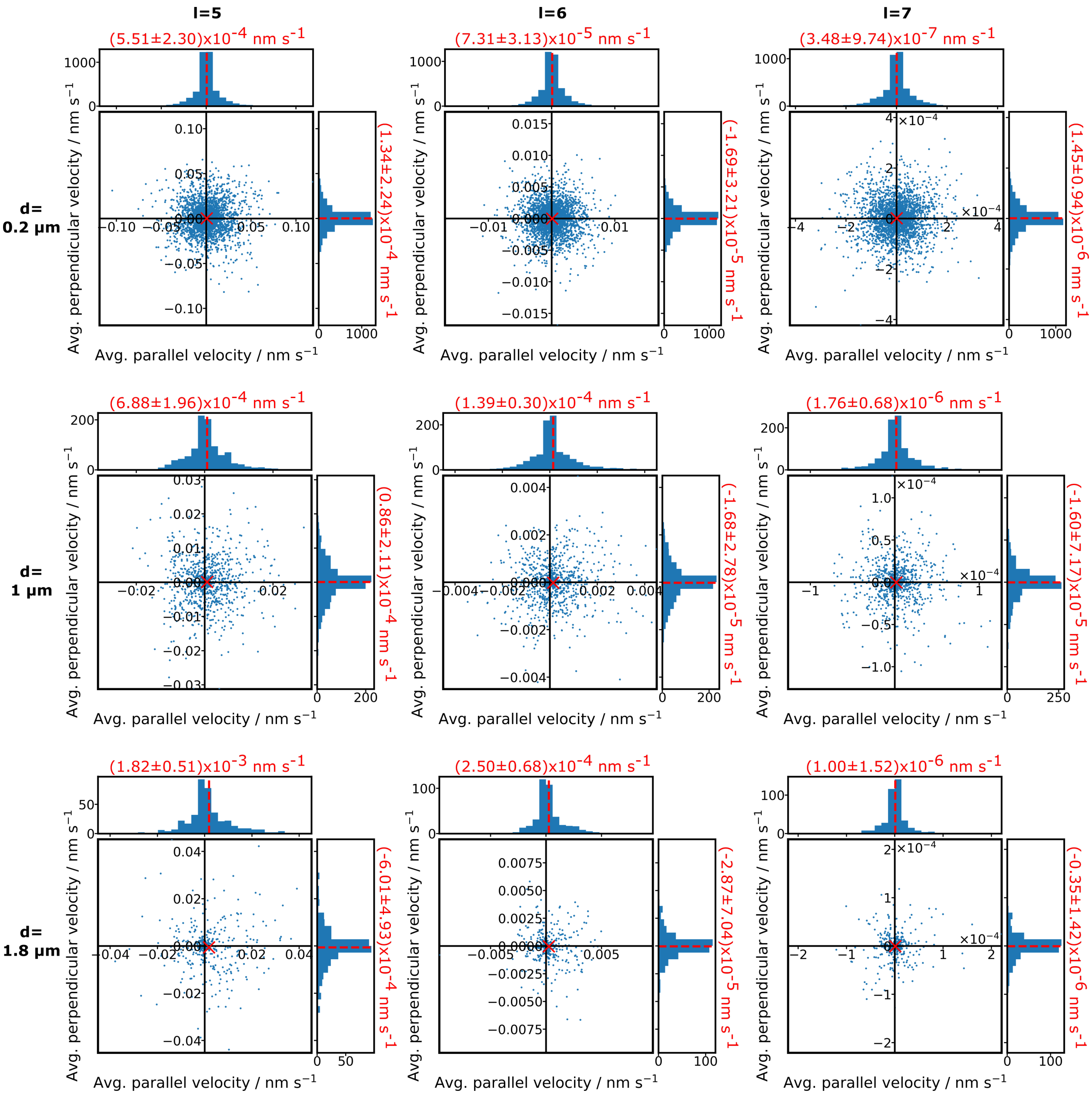

Figure S15: **Full distributions of simulated vesicle velocities relative to the gradient, for varying vesicle diameter and sticky end length.** The plots illustrate simulation results for vesicle velocity parallel and perpendicular to the gradient direction, where the velocity has been calculated as total displacement over the trajectory divided by the total duration. The data is for $l = 5$, 6, and 7 nt sticky end systems, with vesicle diameters $d = 0.2$, 1 and 1.8 μm. Positive values of displacement parallel to the gradient indicate vesicle motion in the direction of increasing DNA surface density on the substrate. The averages of the distributions are marked by red dashed lines and crosses. Data for $d = 1\,\mu$m are also shown in Fig. 2. Note that the GUV counts per velocity bin differ between the histograms shown here and those in Fig. 2 of the main text. This is a consequence of using an equal number of bins in the two figures over a different range of velocities.

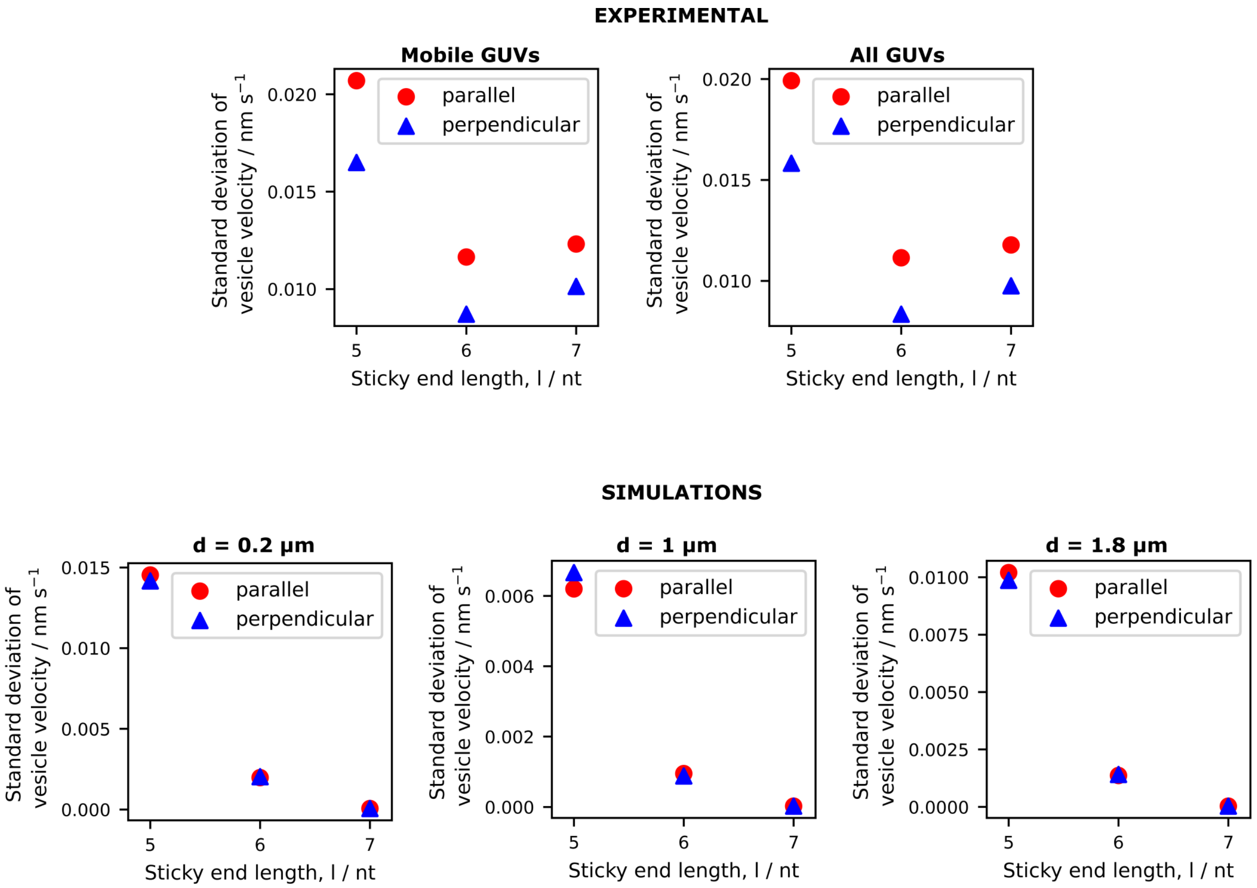

Figure S16: **Standard deviation of vesicle velocities versus sticky end length.** Experimental data (top) are shown for the filtered (mobile) vesicle population (top left) and for all vesicles (top right); the filtering method is outlined in Section 1.4 of the SI. Simulation data are shown for vesicles with diameter $d = 0.2\,\mu$m, $1\,\mu$m and $1.8\,\mu$m.

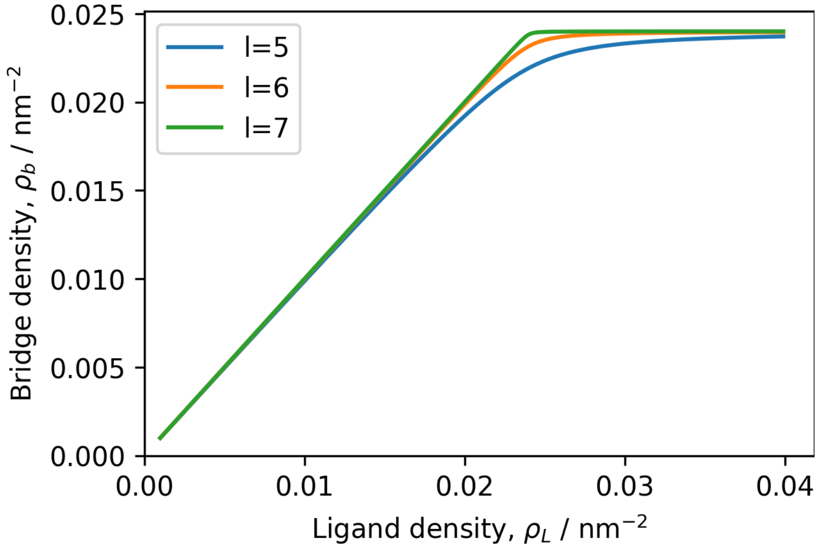

Figure S17: **Under experimental conditions, the density of receptor-ligand bridges is saturated and reaches its maximal value.** The bridge density ρ_{b} is plotted against ligand density ρ_L, while the initial density of receptors over the vesicle membrane ρ_{R} is fixed at $0.008\,\mathrm{nm}^{-2}$, for $0.2\,\mu$m diameter vesicles and sticky ends lengths $l = 5$, 6 and 7 nt (DNA sequences shown in Table S1). The bridge density has been estimated using Eq. 30, with the equilibrium constant K_{eq} estimated using the Arrhenius equation $K_{eq} = \frac{e^{-\beta\Delta_G}}{\rho_0 L A_{tot}}$ where Δ_G is the standard hybridisation energy of the sticky ends, $\rho_0 = 1\,\mathrm{M}$ is the standard concentration, L is the length of the sticky ends and A_{tot} is the total surface area of the vesicle. While $\rho_L < 3\rho_{\mathrm{R}}$ the bridge density is limited by ρ_L, whereas when $\rho_L > 3\rho_{\mathrm{R}}$ the bridge density saturates at $3\rho_L$. The factor of 3 results from the redistribution of the receptors from an initial uniform concentration across the vesicle (approximated as a hemisphere) to being concentrated within the contact region (a third of the total surface area of the hemisphere).

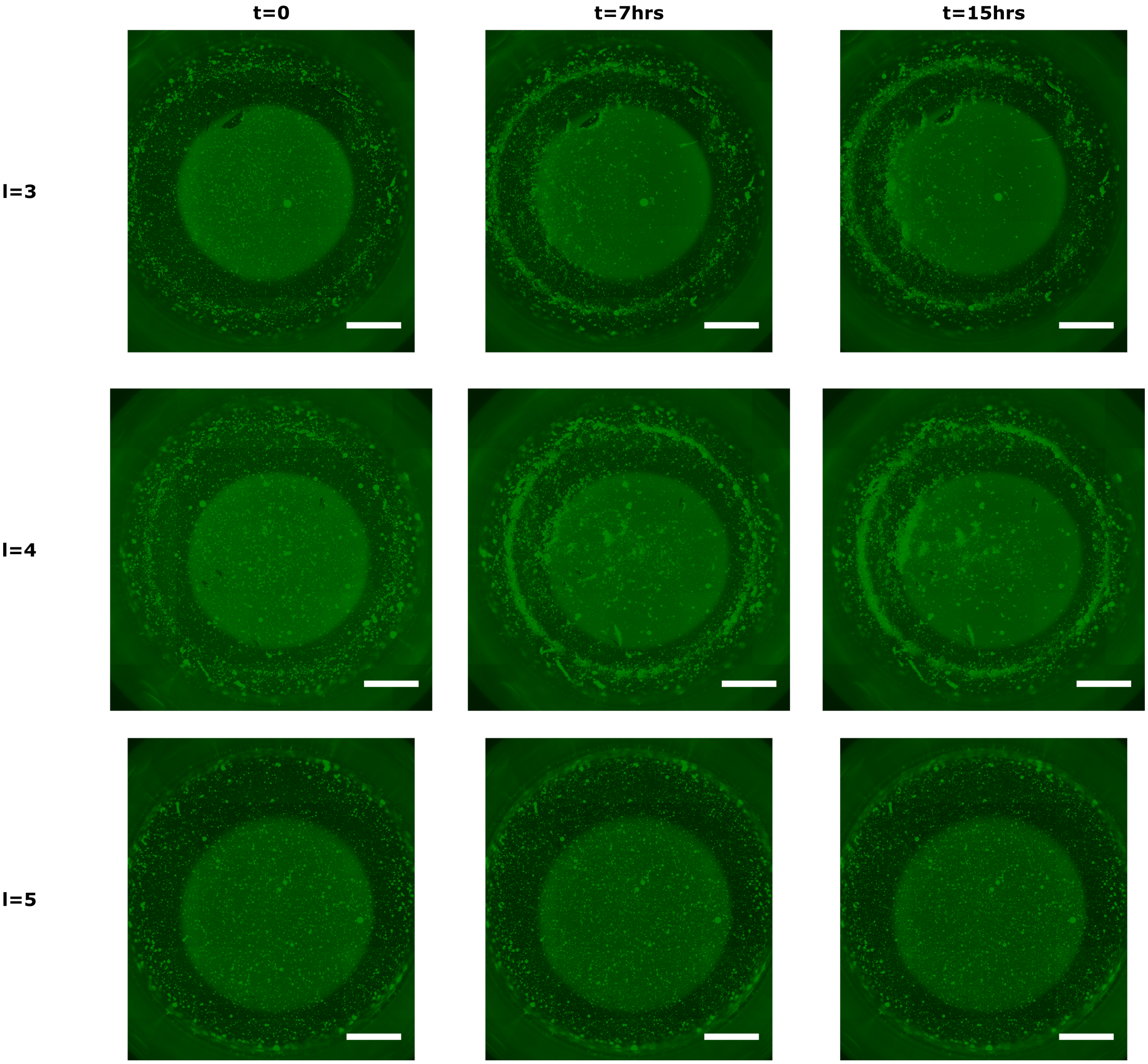

Figure S18: **Effects independent of surface adhesion cause vesicles to globally drift towards one side of the well in weakly adhering systems.** Microscopy images are shown for systems with sticky end lengths $l = 3$–$5\,\mathrm{nt}$, at time $t = 0$, 7 and 14 hours after the experiment started. For the weakly binding systems $l = 3$ and 4, the majority of vesicles drift in one direction and move distances comparable to the width of the well. Scale bars correspond to a length of 1 mm.

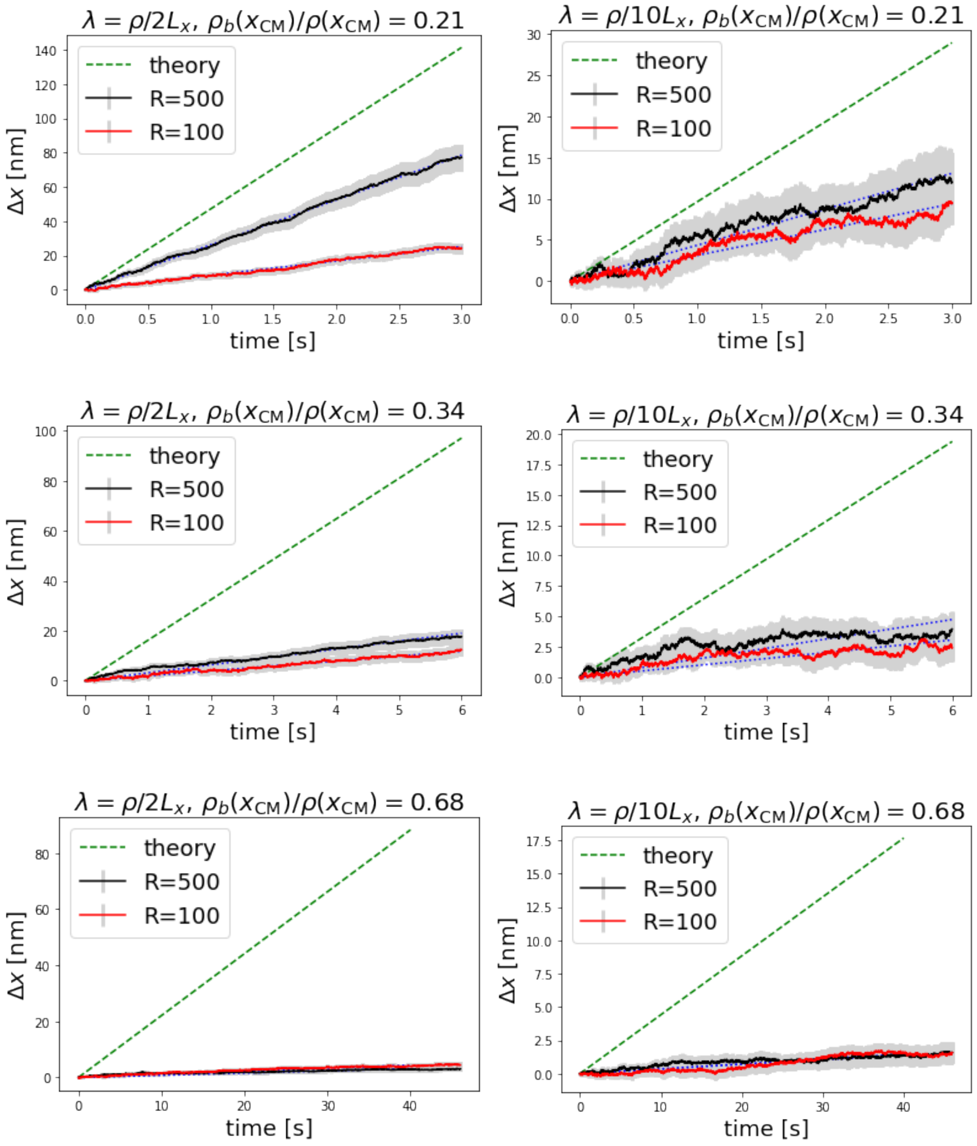

Figure S19: **Comparison of simulated and theoretical vesicle displacements over time, parallel to the gradient, for varying gradients and bridge densities.** Mean displacements for varying gradients (λ) and density of receptor-ligand bridges (ρ_b) predicted by simulations and by theory (Eq. 14) are shown. In each plot, simulation results for two vesicle sizes (radius R =100 nm and 500 nm) are displayed, and straight lines have been fitted to them. x refers to the distance along the gradient, and $\rho(x_{CM})$ is the ligand density within the contact region between the vesicle and substrate. $\rho = 0.021\,\mathrm{nm}^{-2}$ is the reference ligand density, while and L_x are the reference density and gradient length scale. The bridge density has been tuned by varying the off rates of the sticky ends. We find that when increasing ρ_b the gap between simulation and theoretical predictions drastically increases.

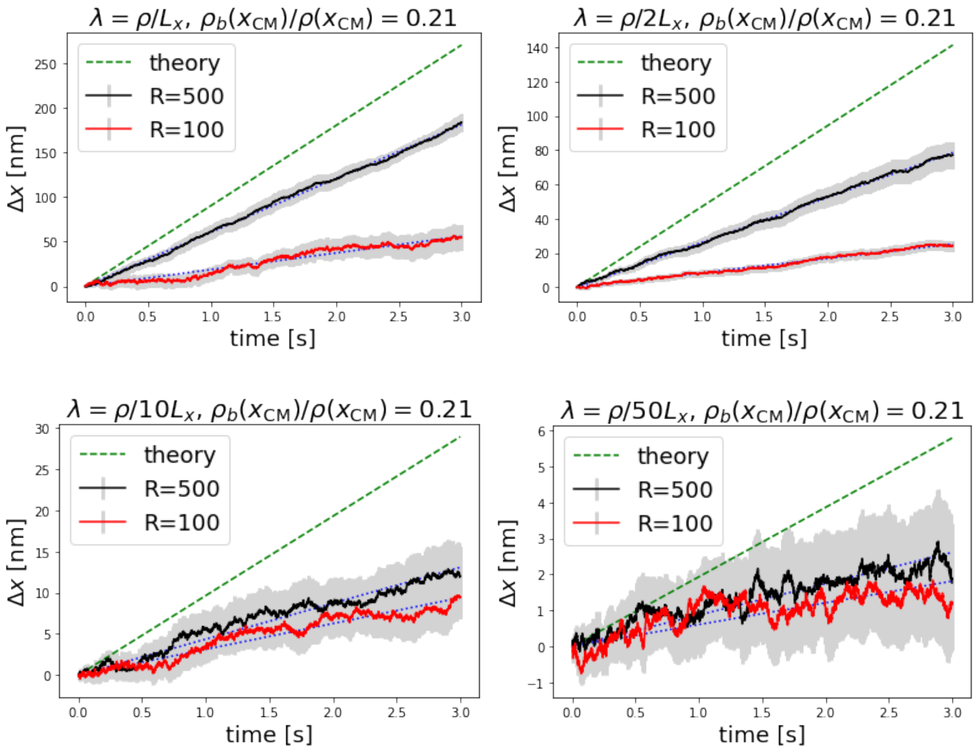

Figure S20: **Comparison of simulated and theoretical vesicle displacements over time, parallel to the gradient, for varying gradients.** Mean average displacements for different gradients (λ) as predicted by simulations of vesicles with two different radii (R), and by theory (Eq. 14 in the main text). $\rho = 0.021\,\mathrm{nm}^{-2}$ is the reference ligand density, while $L_x = 10\,\mu\mathrm{m}$ is the reference gradient length scale. For small values of λ the gap between theory and simulations decreases, and the drifting velocities no longer appear to depend on vesicle size (in agreement with Eq. 14).

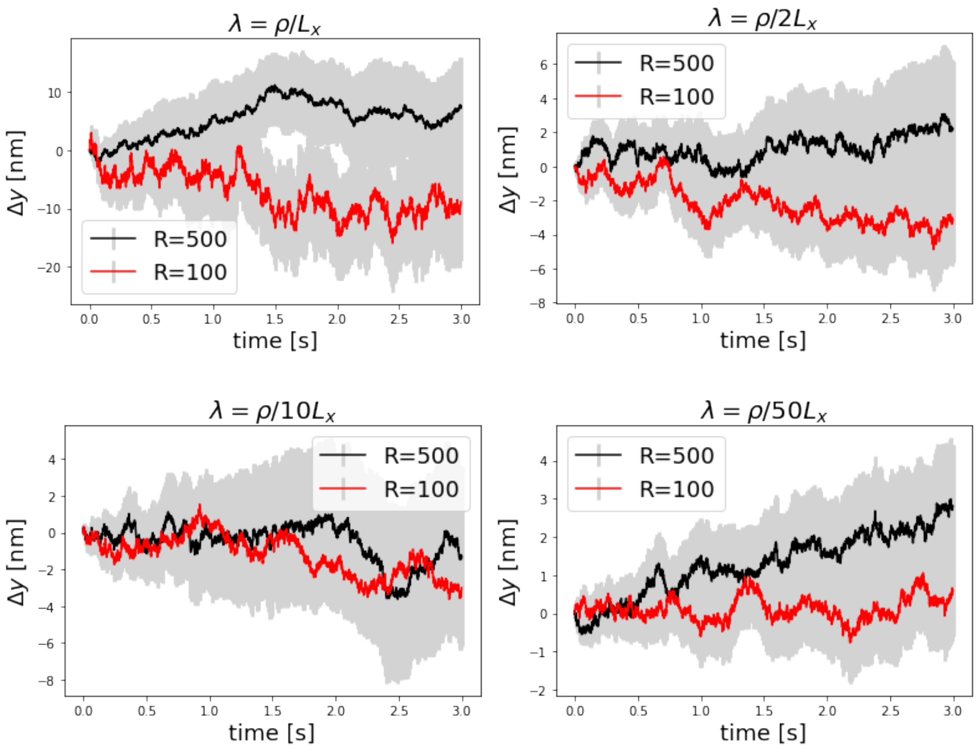

Figure S21: **Comparison of simulated vesicle displacements over time, perpendicular to the gradient, for varying gradients.** Mean average displacements for different gradients (λ) as predicted by simulations of vesicles with two different radii (R). $\rho = 0.021\,\mathrm{nm}^{-2}$ is the reference ligand density, while $L_x = 10\,\mu\mathrm{m}$ is the reference gradient length scale. The bridge density has been tuned to the same density as for the results shown in Fig S20 ($\rho_b(x_{\mathrm{CM}})/\rho(x_{\mathrm{CM}}) = 0.21$) by varying the off rates of the sticky ends.

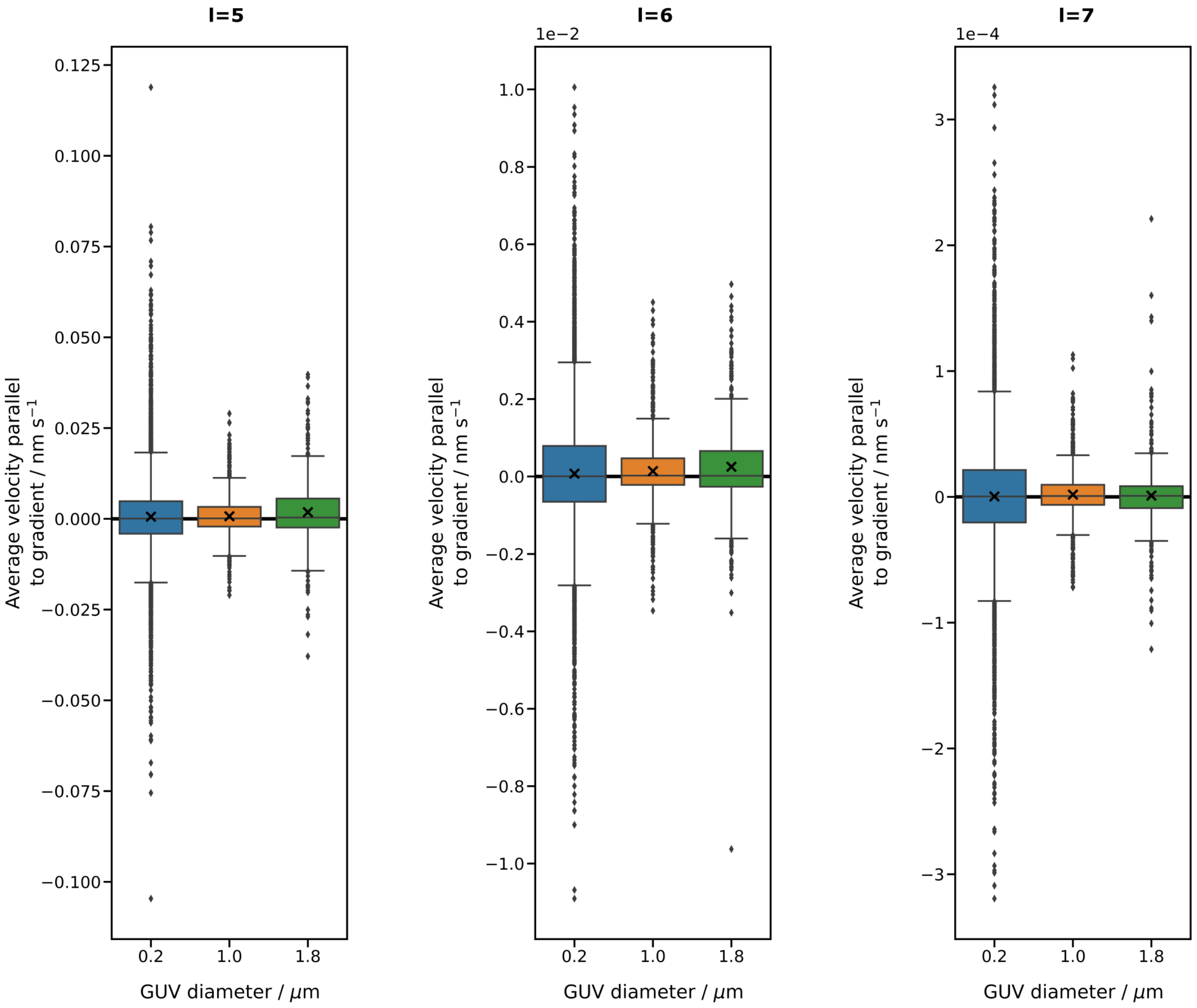

Figure S22: **Distributions of average vesicle velocity parallel to the gradient for 0.2 μm, 1 μm and 1.8 μm diameter vesicles, from simulations.** Data for $l = 5$–7 nt sticky end systems are plotted, including all outliers. Data are shown excluding outliers in Fig. 3. The average vesicle velocities have been calculated as total displacement parallel to the gradient divided by the trajectory duration; positive values of velocity indicate motion along the gradient in the direction of increasing ligand density on the substrate. The mean of the distributions for each vesicle size range is marked with a cross.

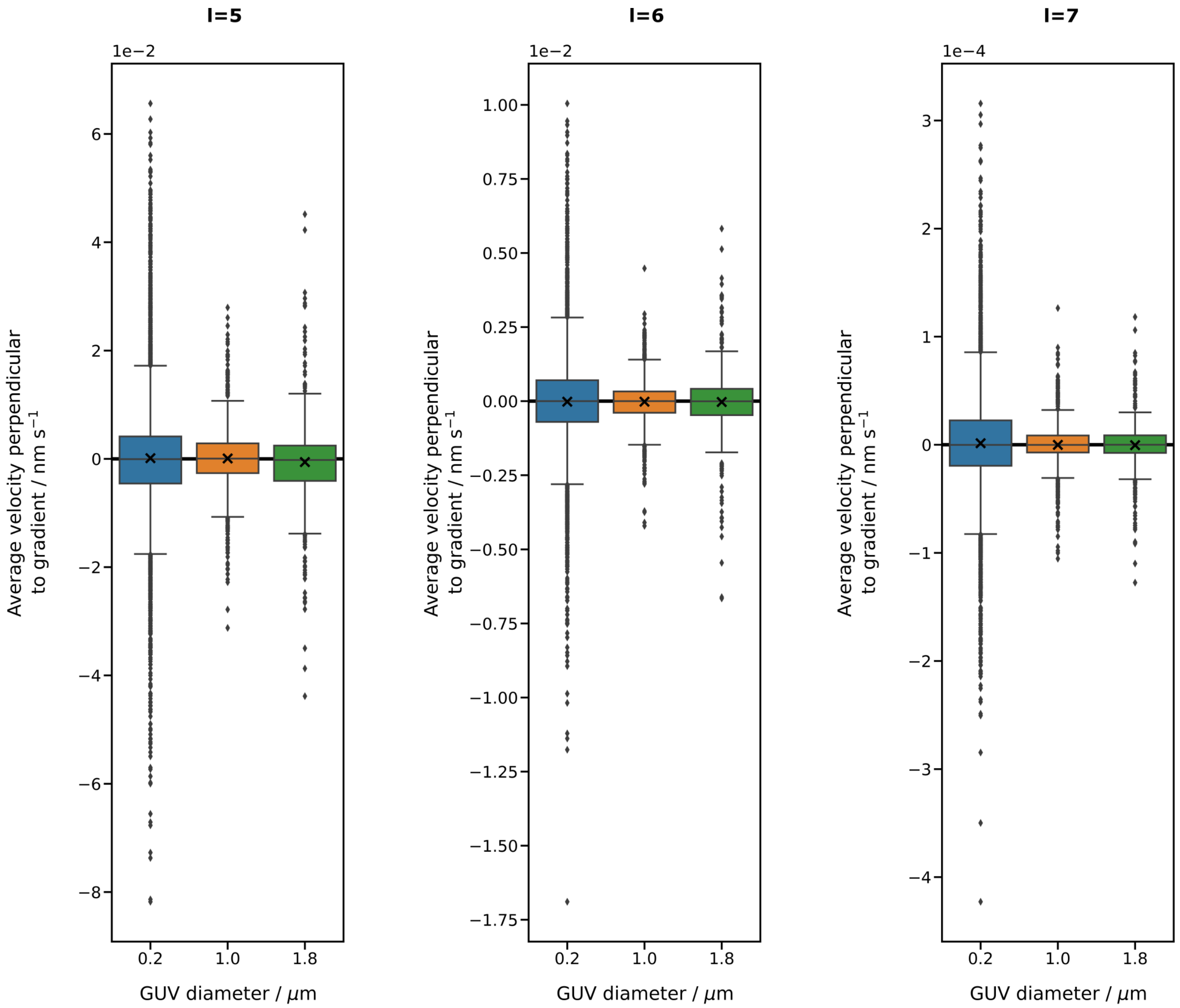

Figure S23: **Distributions of average vesicle velocity perpendicular to the gradient for 0.2 μm, 1 μm and 1.8 μm diameter vesicles, from simulations.** Data for l = 5–7 nt sticky end systems are plotted, including all outliers. The average vesicle velocities have been calculated as total displacement perpendicular to the gradient divided by the trajectory duration. The mean of the distributions for each vesicle size range is marked with a cross.

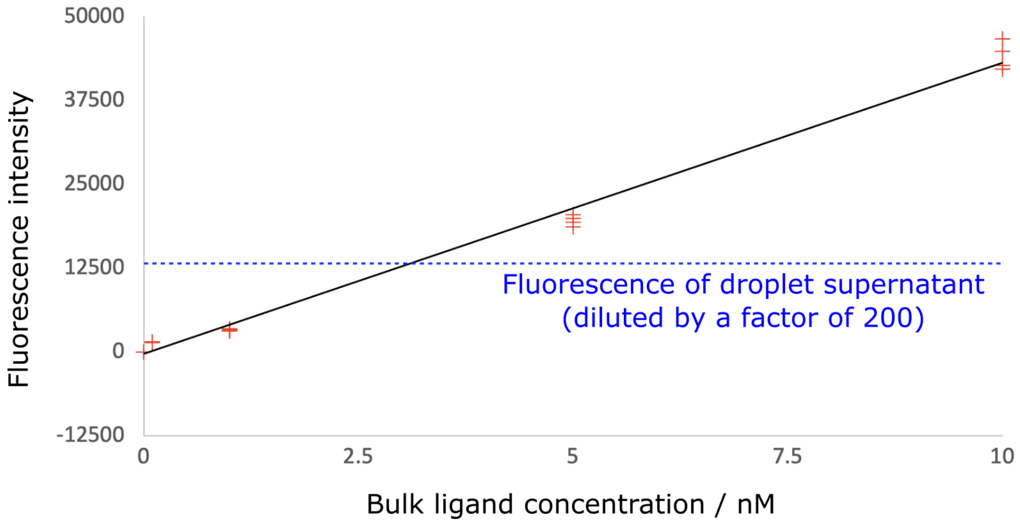

Figure S24: **Calibration curve for determining ligand density from fluorescence signal.** This calibration curve was used to determine an estimate of the ligand density in the maximum density regions of wells in experiments, as outlined in the Materials and Methods section in the main text. The plot shows the background-subtracted fluorescence signals of samples with known ligand concentrations (0, 0.1, 1, 5 and 10 nM) in buffer solution (100 mM NaCl and 1×TE). A straight line has been fitted to the fluorescence measurements to establish a calibration curve. The mean fluorescence intensity of the supernatant of a droplet of ligand solution applied to a well (subsequently diluted by a factor of 200) is indicated with a blue dashed line.

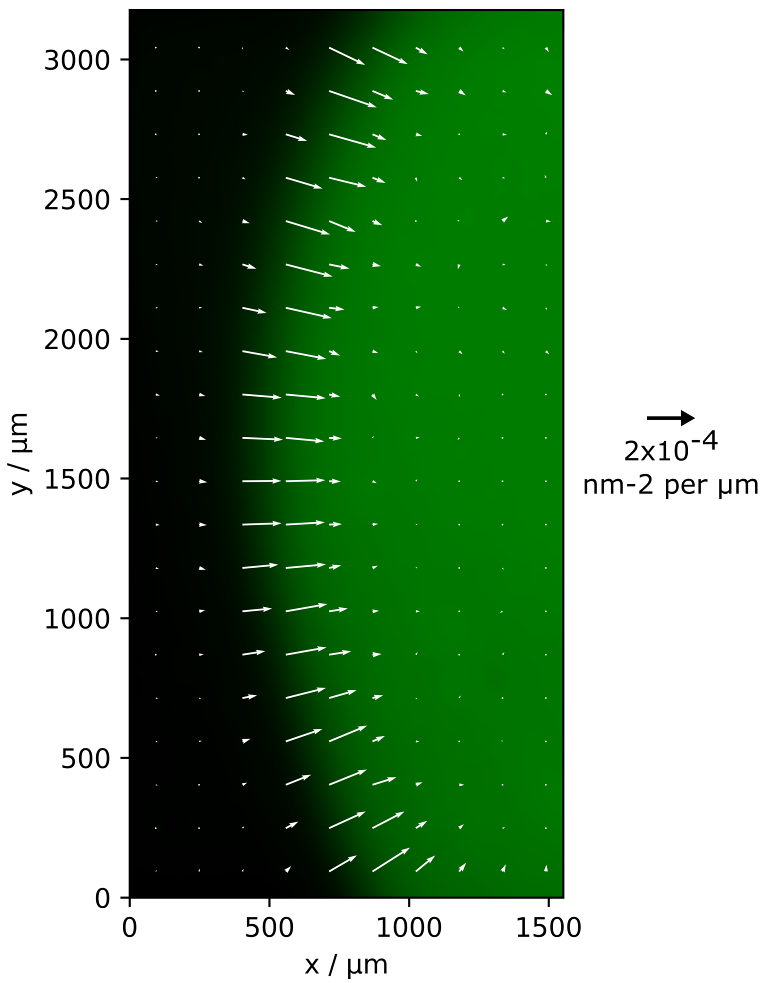

Figure S25: **Plot of the DNA gradient vector field superimposed on a fluorescence image of the ligand coverage.** The fluorescence image of the ligand coverage was recorded *via* epifluorescence microscopy, exciting the Alexa Fluor 488 modifications on the ligand constructs attached to the substrate. The intensity of the green signal corresponds to the local density of ligands. The arrows indicate the local directions of the ligand density gradient at evenly spaced points across the image. The arrow lengths are proportional to the local magnitudes of the ligand gradient. A scale bar for the magnitude of the ligand density gradient is shown to the right of the image.

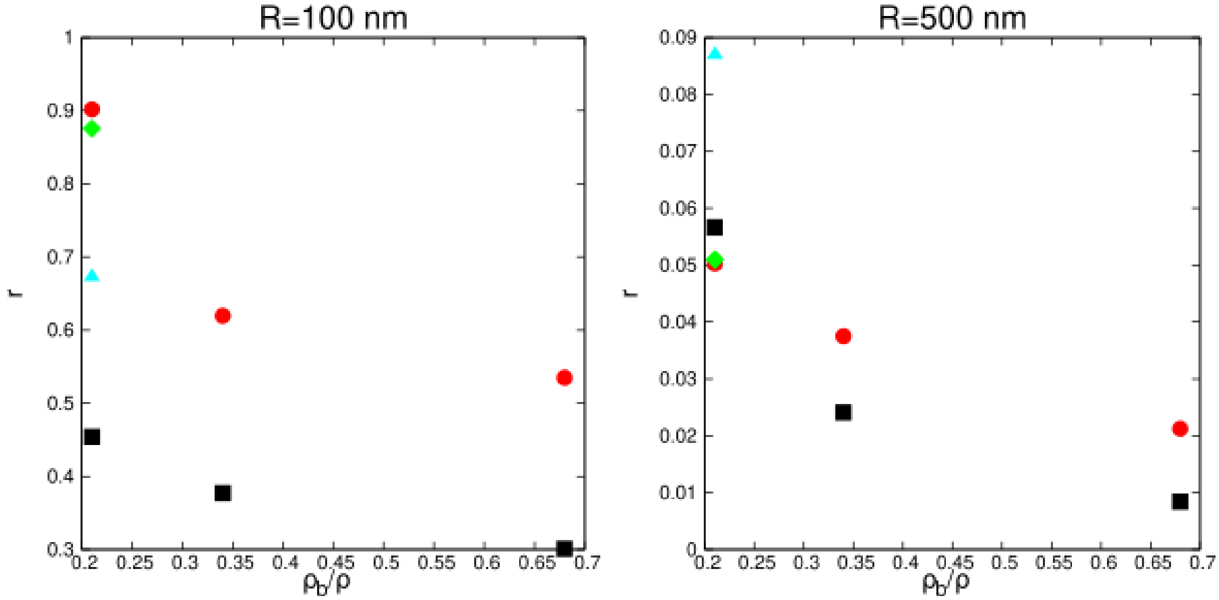

Figure S26: **Deviations of the simulations trajectories from the Langevin equation (Eq. 2).** We report the estimations of r ($r = \delta_{\mathrm{the}}/\delta_{\mathrm{sim}}$, see Sec. 2.3) as a function of the fraction of ligands underneath the vesicle forming bridges, ρ_{b}/ρ. We employed the simulation results of Figs S19 and S21. δ is the ratio between the diffusion constant and the drifting velocity. For trajectories following the Langevin equation (Eq. 2), one would expect $r = 1$. Triangles, squares, circles, and diamonds correspond, to gradient's slopes (λ) equal to, respectively, ρ/L_x, $\rho/2L_x$, $\rho/10L_x$, and $\rho/50L_x$. The two panels refer to two different vesicle sizes ($R = 100\,\mathrm{nm}$ and $R = 500\,\mathrm{nm}$).

References

(1) Li, K. The image stabilizer plugin for ImageJ. `http://www.cs.cmu.edu/~kangli/code/Image_Stabilizer.html`, 2008; Published: 2008-02-01.

(2) Fukai, Y. T.; Kawaguchi, K. LapTrack: Linear Assignment Particle Tracking with Tunable Metrics. *Bioinformatics* **2022**, *39*, btac799.

(3) Fukai, Y. T. laptrack. 2021; `https://doi.org/10.5281/zenodo.5519537`.

(4) Einstein, A. Über die von der molekularkinetischen Theorie der Wärme geforderte Bewegung von in ruhenden Flüssigkeiten suspendierten Teilchen. *Annalen der physik* **1905**, *4*.

(5) Von Smoluchowski, M. Zur kinetischen theorie der brownschen molekularbewegung und der suspensionen. *Annalen der physik* **1906**, *326*, 756–780.

(6) Hamming, P. E.; Overeem, N. J.; Diestelhorst, K.; Fiers, T.; Tieke, M.; Vos, G. M.; Boons, G.-J. P.; van der Vries, E.; Block, S.; Huskens, J. Receptor Density-Dependent Motility of Influenza Virus Particles on Surface Gradients. *ACS applied materials & interfaces* **2023**, *15*, 25066–25076.

(7) Lowensohn, J.; Stevens, L.; Goldstein, D.; Mognetti, B. M. Sliding across a surface: particles with fixed and mobile ligands. *The Journal of Chemical Physics* **2022**, *156*, 164902.

(8) Mognetti, B. M.; Cicuta, P.; Di Michele, L. Programmable interactions with biomimetic DNA linkers at fluid membranes and interfaces. *Reports on progress in physics* **2019**, *82*, 116601.

(9) Lau, A. W.; Lubensky, T. C. State-dependent diffusion: Thermodynamic consistency and its path integral formulation. *Physical Review E* **2007**, *76*, 011123.

(10) Widder, C.; Koch, F.; Schilling, T. Generalized Langevin dynamics simulation with non-stationary memory kernels: How to make noise. *The Journal of Chemical Physics* **2022**, *157*.

(11) Netz, R. R. Derivation of the non-equilibrium generalized Langevin equation from a generic time-dependent Hamiltonian. 2023.

(12) Rice, J. A. *Mathematical statistics and data analysis*; Thomson/Brooks/Cole Belmont, CA, 2007; Vol. 371.
